# Integrating Network Pharmacology and Experimental Validation to Elucidate the Mechanism of Jiegeng Decoction in Improving Allergic Asthma

**DOI:** 10.1021/acsomega.3c06914

**Published:** 2023-12-06

**Authors:** Zhihai Wu, Zhiqiang Luo, Wen Sun, Yuanyuan Shi, Qi Ding

**Affiliations:** † School of Life Sciences, Beijing University of Chinese Medicine, Beijing 100029, China; ‡ National Key Laboratory for Quality Ensurance and Sustainable Use of Dao-di Herbs, Beijing 100700, China; § State Key Laboratory of Dao-di Herbs, National Resource Center for Chinese Materia Medica, China Academy of Chinese Medical Sciences, Beijing 100700, China; ∥ Shenzhen Research Institute, Beijing University of Chinese Medicine, Shenzhen 518118, China

## Abstract

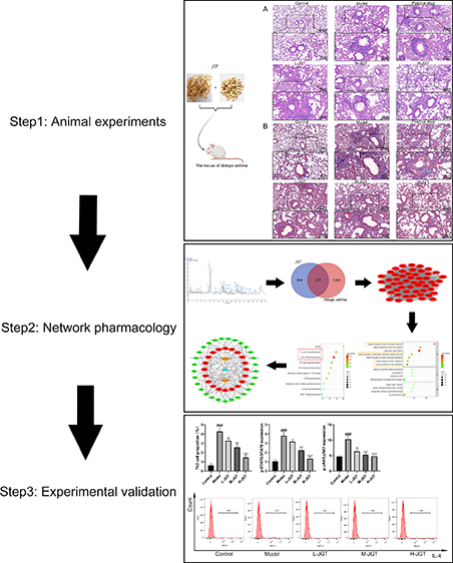

Allergic asthma is a prevalent form of asthma that is characterized primarily by airway inflammation. Jiegeng decoction (JGT) is a traditional Chinese herbal formula known for its anti-inflammatory properties and has been used to treat respiratory diseases for centuries. This study aimed to investigate the biological effects and mechanisms of action of JGT in improving allergic asthma. An experimental allergic asthma mouse model was established using ovalbumin. The results showed that JGT significantly improved inflammation cell infiltration in the lung tissue of allergic asthmatic mice and the inflammatory environment of Th2 cells in the bronchoalveolar lavage fluid while also reducing serum IgE levels. Subsequently, 38 components of JGT were identified through liquid chromatography–mass spectrometry. Network pharmacology revealed that regulating inflammation and immune responses is the primary biological process by which JGT improves allergic asthma, with Th2 cell differentiation and the JAK-STAT signaling pathway being the key mechanisms of action. Finally, qPCR, flow cytometry, and Western blotting were used to validate that JGT inhibited Th2 cell differentiation by blocking the JAK1-STAT6 signaling pathway in CD4^+^ T cells, ultimately improving allergic asthma. This study provides a novel perspective on the therapeutic potential of JGT in the treatment of allergic asthma.

## 1 Introduction

Asthma is a persistent respiratory condition characterized by inflammation of the airways.^1^ According to the Global Burden of Disease Report, asthma has been assessed as the most prevalent among chronic respiratory disorders, affecting approximately 358 million individuals.^2^ Among the different forms of asthma, allergic asthma emerges as the most frequently encountered. Currently, antitype 2 biologics, including omalizumab and mepolizumab, are the primary drugs used to treat allergic asthma by targeting effector cells and cytokines.^3^ Despite the improved quality of life experienced by many patients with allergic asthma owing to the use of antitype 2 biologics, clinical trials have shown that a significant number of patients do not respond favorably to these medications.^4^ Therefore, it is imperative to identify efficacious medications targeting the fundamental pathological mechanisms responsible for allergic asthma.

T helper type 2 (Th2) cell responses, which are linked with allergic asthma, can be initiated by environmental allergens such as house dust mites during early life as well as later-life exposure to novel allergens encountered in occupational settings. Once the allergen is identified, Th2 cells can release type 2 cytokines, such as interleukin (IL)-4, IL-5, and IL-13, resulting in the recruitment of eosinophils (EOS) to the airway wall and the synthesis of allergen-specific immunoglobulin E (IgE) by B cells. When they bind to the allergen, two neighboring IgE molecules connect, triggering the activation of mast cells and basophils, leading to the release of biologically active mediators such as histamine. These processes collectively contribute to bronchial hyper-responsiveness (BHR). Consequently, therapeutic strategies addressing multiple pathways and targets hold the potential for enhancing the management of allergic asthma.^4^

Traditional Chinese medicine (TCM) formulas achieve therapeutic effects through the synergistic activity of multiple compounds, targets, and pathways.^4,5^ Jiegeng decoction (JGT) is composed of a 1:2 ratio of *Platycodon grandiflorus* (Jacq.) A.DC. (known as Jiegeng in Chinese) and *Glycyrrhiza uralensis* Fisch. ex DC. (known as Gancao in Chinese). It was first mentioned in the Chinese classical medical book Treatise on Typhoid and Miscellaneous Diseases.^6^ JGT promotes lung function, removes phlegm, and exerts anti-inflammation effects and is regarded as a crucial TCM formula for treating lung diseases.^7^ However, no studies have investigated the biological effects and mechanisms of action of JGT for treating allergic asthma.

This study first explored the therapeutic potential of JGT in treating allergic asthma. Subsequently, the chemical composition of JGT underwent analysis using liquid chromatography–mass spectrometry (LC-MS). Utilizing network pharmacology, our aim encompassed unveiling essential targets, primary biological processes, and potential pathways contributing to JGT’s advantageous effects on allergic asthma. Subsequently, experimental validation was undertaken to substantiate the predictive findings derived from the network pharmacology analysis.

## 2 Materials and Methods

### 2.1 Animal Experiments

#### 2.1.1 Drug Preparation

*Platycodon grandiflorus* (Jacq.) A.DC. (Jiegeng in Chinese) was purchased from Anhui Guanghe Traditional Chinese Medicine Co., Ltd. (batch number 201208); *Glycyrrhiza uralensis* Fisch. ex DC. (Gancao in Chinese) was purchased from Lingnan Traditional Chinese Medicine Slices Co., Ltd. (batch number 2104002). The two plants mentioned above were verified through World Flora Online. The quality of the medicinal materials was confirmed by Dr. Qi Ding. The plants were weighed in proportion to the JGT recipe. The plant mixture was immersed in pure water at five times its weight, followed by boiling for 1 h, and finally filtered to isolate the filtrate and residue. This process was repeated by adding 2.5 times its weight to purified water and boiling for another hour followed by the same filtration step. The filtrate was combined and concentrated using a rotary evaporator, and the resulting solution was stored in a refrigerator at 4 °C for in vivo experiments.

#### 2.1.2 LC-MS Analysis

A Dionex Ultimate 3000 UHPLC system and a Q-Exactive Orbitrap mass spectrometer, both manufactured by Thermo Fisher Scientific in the United States, were employed. Sample separation was achieved using an Agilent ZORBAXSB-C18 column (50 × 4.6 mm inner diameter, 1.8 μm). We used the following gradient elution conditions: 0–5 min with 6% acetonitrile, 5–15 min with 40% acetonitrile, and 15–35 min with 100% acetonitrile. The flow rate was set at 0.6 mL/min while maintaining the column temperature at 30 °C. Negative ion mode mass spectrometry analysis was conducted with a spray voltage set at −3 kV and a capillary temperature maintained at 350 °C. The mass spectrum was scanned in the range of 150 to 1500 *m*/*z*. Thermo Xcalibur software (ver. 4.0) was used to collect and analyze data.

#### 2.1.3 Animals and Grouping

A total of 60 female BALB/c mice, aged 7 weeks and weighing between 18 and 20 g, were procured from Zhuhai BesTest Bio-Tech Co., Ltd. (Zhuhai, China). A 7-day acclimatization period was implemented to facilitate the mice’s adaptation to the laboratory environment, ensuring their physiological equilibrium in preparation for the study. The mice were allocated in a random manner to six distinctive groups, namely, the control, model, budesonide, L-JGT, M-JGT, and H-JGT groups. During the experiment, the mice were housed in a meticulously controlled setting, with a temperature maintained at 22 ± 2 °C, a relative humidity of 55 ± 2%, and an alternating light–dark cycle of 12 h each. Furthermore, the mice had unrestricted access to standard chow and water. Conformity to the National Institute of Health Guide for the Care and Use of Laboratory Animals was ensured for all experimental protocols, and ethical clearance was obtained from the Animal Ethical Committee of Shenzhen Research Institute, Beijing University of Chinese Medicine (approval number SZI-AE-2023030101).

#### 2.1.4 Mouse Model and Treatment

**Figure 1:**
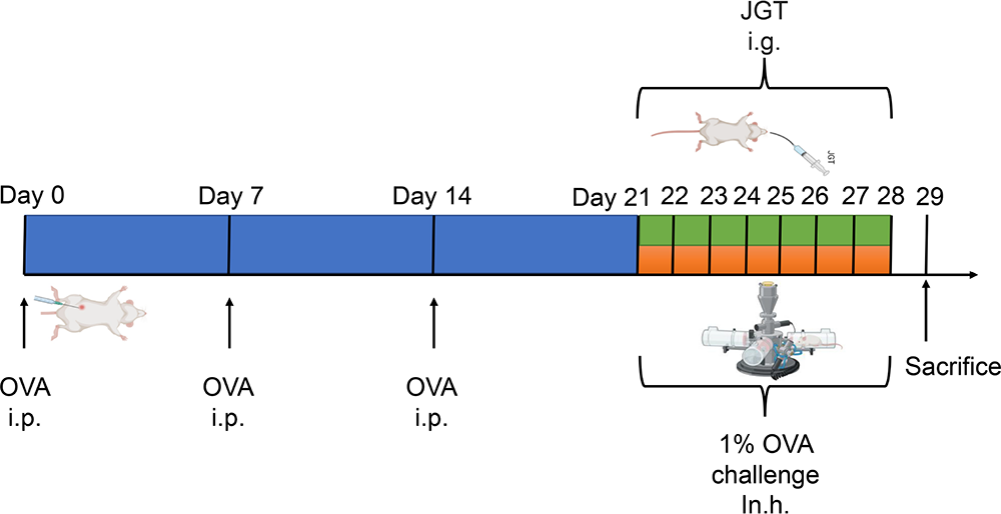
Development of a mouse model of allergic asthma and treatment. i.p. and blue represent intraperitoneal injection, i.g. and green represent oral gavage, and In.h. and orange represent inhalation.

The allergic asthma mouse model was prepared using all animals except those in the control group. The sensitization phase encompassed a series of intraperitoneal injections, administered on days 0, 7, and 14. Specifically, during each injection, 0.2 mL of a sterile saline solution was introduced into the peritoneal cavity of each individual murine subject. This solution consisted of 50 μg of ovalbumin (OVA) (Sigma) and 0.05 mL of adjuvant alum (Aladdin). The intent of this procedure was to induce an immune sensitization response in the mice toward the OVA. Subsequent to the final sensitization regimen on day 21, the murine subjects were introduced into a specially designed aerosol exposure system. Over the ensuing 7-day period, these subjects were subjected to daily inhalation of aerosolized 1% OVA, wherein each exposure session spanned a duration of 30 min. The treated mice were categorized into control, model, and positive drug groups (budesonide, 0.2 mg/mL/day) and JGT treatment groups with varying doses, including low-dose (L-JGT, 5.87g/kg/day), middle-dose (M-JGT, 11.74g/kg/day), and high-dose (H-JGT, 23.48g/kg/day). During days 21 to 27, the treatment groups were administered JGT once daily via oral gavage (Figure 1). The mice in the positive drug group were treated with aerosol inhalation. The control group of mice was challenged and treated with an equivalent volume of normal saline.

#### 2.1.5 Lung Histopathology

This study focused on a comprehensive exploration of the microstructural characteristics and potential features of the lungs and trachea through detailed histological analysis. Initially, a 4% paraformaldehyde solution (Biosharp, Shanghai, China) was applied for the fixation of lung and trachea specimens for an extended period of 48 h, ensuring the stability of cellular and tissue structures. Subsequently, the specimens underwent paraffin embedding and sectioning, establishing a dependable foundation for subsequent staining procedures. Regarding the choice of staining methods, the selection encompassed hematoxylin and eosin (H&E) staining along with Masson’s staining.

#### 2.1.6 Collection of the Serum and Bronchoalveolar Lavage Fluid (BALF)

Blood samples were meticulously drawn from the conjunctival plexus of the experimental animals. Subsequently, a centrifugation protocol was applied to the blood samples, spinning them at a speed of 3000 rpm for 15 min, resulting in the essential serum component. At the same time, the animals underwent a lung lavage procedure, during which the lungs were washed three times with 1 mL of sterile phosphate-buffered saline (PBS) (Gibco, New York) each time. Moving forward, the collected BALF was subjected to another centrifugation step at 1500 rpm for 10 min, executed under controlled conditions at 4 °C, culminating in the isolation of the precipitate. The ensuing precipitate was subsequently reconstituted in PBS, and the ensuing cellular populace was meticulously categorized and quantified utilizing Diff Quik staining (Solarbio, Beijing, China). To delve into the realm of cytokine assessment, both the supernatants derived from the cellular suspension and the serum were judiciously preserved, maintaining a temperature of −80 °C to ensure the stability of the samples for ensuing analyses. Ultimately, enzyme-linked immunosorbent assay (ELISA) kits (Biolegend, Beijing, China; Proteintech, Wuhan, China) were wielded as the analytical tool of choice, adhering closely to the guidelines prescribed by the manufacturer.

#### 2.1.7 Sorting of CD4^+^ T Cells

The spleens of the mice were sliced into small pieces, ground using a grinder, and filtered through a 200-mesh filter (Biosharp, Shanghai, China). Lymphocytes were obtained by using a lymphocyte separation solution (Dayou, Shenzhen, China). CD4^+^ T cells were isolated from lymphocytes by magnetic bead sorting (Biolegend, Beijing, China).

#### 2.1.8 Quantitative Real-Time Polymerase Chain Reaction

A sequence of standardized experimental procedures was employed to assess the expression levels of specific genes within the lung tissue and CD4^+^ T cells. Initial steps encompassed the extraction of the total RNA from the target samples, providing the requisite nucleic acid material for subsequent analyses. RNA extraction was executed utilizing an RNA extraction kit (TIANGEN BIOTECH, China), facilitating the efficient purification of RNA from both the lung tissue and CD4^+^ T cells. Subsequently, cDNA synthesis was conducted utilizing a reverse transcription kit (Vazyme Biotech, China). To profile gene expression, we utilized quantitative real-time polymerase chain reaction (qPCR) technology. This phase of the investigation was conducted on a Roche LightCycler 480 system. Within the RT-qPCR reactions, a ChamQ SYBR qPCR Master Mix (Vazyme Biotech, China) was employed, delivering essential enzymes and fluorescent probes to facilitate the reactions. To obtain relative expression quantification, the 2^–ΔΔct^ method was employed. The primer sequences are provided in Table 1.

**Table 1:** Target Gene Primer Sequence

**target gene**	**forward primer** (5′→3′)	**reverse primer** (5′→3′)
IL-4	GATGTGCCAAACGTCCTCAC	GAAGCACCTTGGAAGCCCTA
IL-5	CCATGAGCACAGTGGTGAAAG	ACAGGAAGCCTCATCGTCTC
IL-13	ATGCCATCTACAGGACCCAG	GATTTTGGTATCGGGGAGGCT
JAK1	GACCAGGCAAGATCCAGACA	CCCTCTCCCAAGTCACGAAT
STAT6	CATCACCATTGCACACGTCA	TGAGCGAATGGACAGGTCTT
GAPDH	AAATGGTGAAGGTCGGTGTGAAC	CAACAATCTCCACTTTGCCACTG

#### 2.1.9 Flow Cytometry

CD4^+^ T cells were stimulated using a cell activation cocktail with Brefeldin A (Biolegend, Beijing, China) for 4 h, incubated with an antimouse CD4 surface marker (Biolegend, Beijing, China), and then fixed using a Cyto-Fast Fix/Perm Buffer (Biolegend, Beijing, China) to destroy the cell membrane. Following this, CD4^+^ T cells were subjected to staining with antimouse IL-4 APC (Biolegend, Beijing, China). The sample was subsequently analyzed using flow cytometry, and the resulting data were imported into FlowJo v10.8.1 software (BD Life Sciences) for further analysis.

#### 2.1.10 Western Blotting

Protein extraction was conducted using a RIPA lysis buffer (Beyotime, China) along with the addition of both phosphatase and protease inhibitors. Following protein extraction, precise protein concentration measurements were carried out by utilizing a BCA protein quantification kit. Subsequently, protein samples were combined with a 10% SDS-PAGE gel (Solarbio, China) to effect protein separation through gel electrophoresis. Upon completion of the separation process, proteins were transferred onto poly(vinylidene fluoride) (PVDF) membranes to facilitate subsequent immunodetection. To mitigate nonspecific binding, PVDF membranes were subjected to a blocking procedure involving 5% skim milk. During the immunodetection phase, specific antibodies were incubated with the target protein on the PVDF membrane, a process carried out overnight at 4 °C. Afterward, suitable secondary antibodies were used to enhance the signal following the primary antibody reaction. Visualization of protein bands was achieved through the utilization of an ECL chemiluminescence solution (Beyotime), enabling reliable visualization of protein bands. Ultimately, the quantitative analysis of protein bands was performed using ImageJ software.

### 2.2 Network Pharmacology

#### 2.2.1 Determination of Potential Targets of JGT and Allergic Asthma

We identified the targets of the compounds in JGT through utilization of the TCMSP, Swisstarget, and Batman databases. Targets associated with allergic asthma were collected from five databases: OMIM (http://www.omim.org), TTD (https://db.idrblab.org/ttd/), GeneCards (https://www.genecards.org), DrugBank (https://www.drugbank.ca), and CTD (ctdbase.org/). These targets were then verified by importing them into UniProtKB (http://www.uniprot.org).

#### 2.2.2 Constructing of a Protein–Protein Interaction (PPI) Network for Drug Disease Intersection Targets

Utilizing the STRING database (comprehensive score > 0.4), we generated a PPI network diagram highlighting the common targets shared by allergic asthma and JGT. The PPI network diagram was visualized in the Cytoscape 3.2.1 platform. To compute the topological metrics of nodes within the PPI network, we employed the CytoHubba plugin (http://apps.cytoscape.org/cytohubba). Subsequently, we applied the screening criteria that required “betweenness centrality, closeness centrality, and degree” to all be greater than the average, in order to further identify the core targets.

#### 2.2.3 Gene Ontology (GO) and Kyoto Encyclopedia of Genes and Genomes (KEGG) Enrichment Analysis

To elucidate the categorization and functional roles of essential targets within signaling pathways, the DAVID database (https://david.ncifcrf.gov/) was employed for annotating GO functions and performing KEGG pathway analysis on these pivotal targets. The GO functions encompassed molecular functions, cellular components, and biological processes. By implementing a significance threshold of *p* < 0.05, we detected noteworthy GO and KEGG pathways.

#### 2.2.4 Constructing a Component–Target–Pathway Diagram Related to Potential Pathways

To enhance the comprehension of how JGT operates as a treatment for allergic asthma, we selected potential pathways for component–target–pathway visualization. The analysis was conducted using Cytoscape 3.2.1.

### 2.3 Statistical Analysis

Data are represented as the mean accompanied by the standard deviation, and the statistical analysis was conducted using SPSS software (version 17.0). Statistical significance was determined using one-way ANOVA with Tukey’s adjustment, considering *p*-values less than 0.05 as significant. Data visualization and analysis were conducted by using GraphPad Prism 6.

## 3 Results

### 3.1 Determination of the Main Components in JGT through LC-MS Analysis

**Figure 2:**
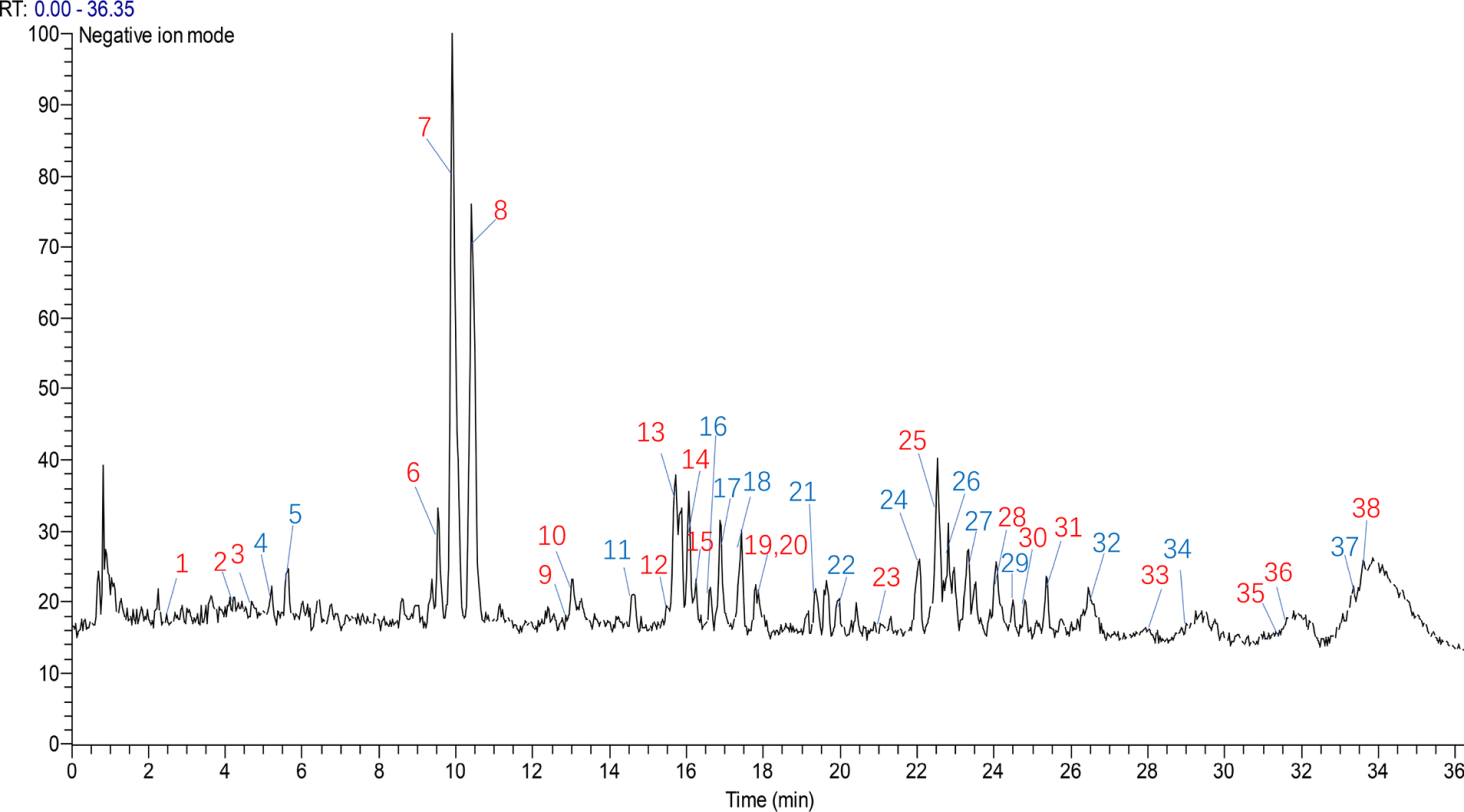
Total ion chromatogram obtained in the negative ionization mode. The red font represents the identified phytochemicals. The blue font represents unidentified phytochemicals.

The main components of JGT were determined by LC-MS analysis. As depicted in Figure 2 and elaborated in Table S1, we identified a comprehensive set of 38 compounds by comparing them with the literature, employing accurate mass measurements, fragmentation rules, MS-MS data, and chromatographic behavior.^8^

### 3.2 JGT Alleviated Airway Inflammation in Allergic Asthma

**Figure 3:**
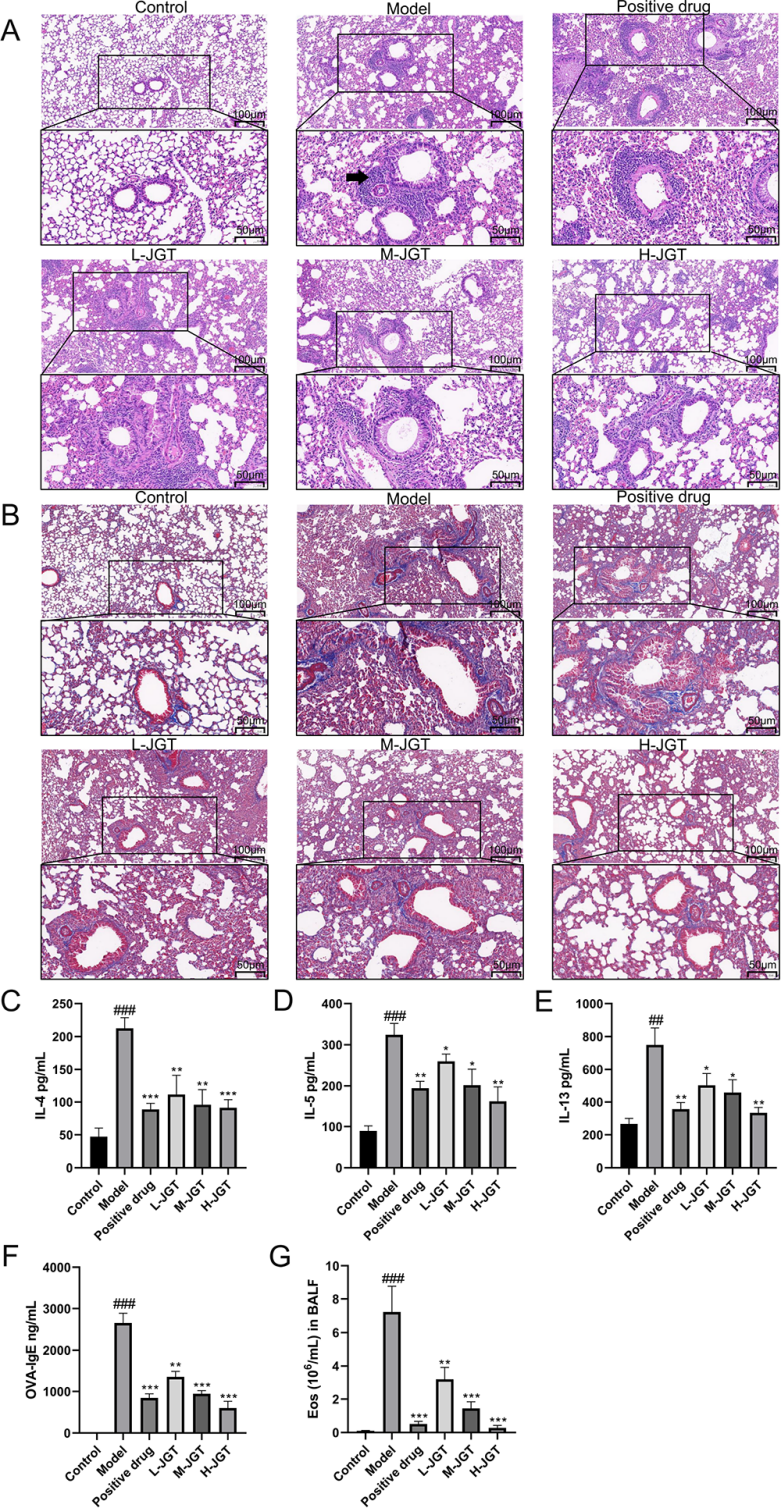
JGT alleviated airway inflammation in allergic asthma. (A) H&E staining of the lung tissue (*n* = 3, scale bars = 50 and 100 μm). The black arrows indicate the inflammatory cell infiltration. (B) Masson’s staining of the lung tissue (*n* = 3, scale bar = 50 and 100 μm). (C–E) Concentrations of IL-4, IL-5, and IL-13 in the BALF (*n* = 3). (F) Concentration of OVA-specific IgE in serum (*n* = 3). (G) Diff Quik staining of the BALF (*n* = 3). ^##^*p* < 0.01, ^###^*p* < 0.001, compared to the control group; *p < 0.05, **p < 0.01, ***p < 0.001, compared to the model group.

Histological examination of the lung tissue was performed utilizing H&E and Masson’s trichrome staining. Consequently, we observed an increase in the infiltration of inflammatory cells in the model group. However, JGT demonstrated noteworthy suppression of pulmonary inflammatory cell infiltration (Figure 3A,B). Furthermore, JGT treatment led to a dose-dependent decrease in the levels of IL-4, IL-5, and IL-13 in BALF. Additionally, JGT treatment also reduced the serum concentration of IgE (Figure 3C–F). Diff Quik staining of the BALF revealed a significant increase in the concentration of EOS in the model group. Conversely, treatment with JGT led to a dose-dependent decrease in the EOS levels in the BALF (Figure 3G). Overall, these findings suggested that JGT could reduce airway inflammation in patients with allergic asthma.

### 3.3 Potential Mechanisms of JGT in the Treatment of Allergic Asthma

**Figure 4:**
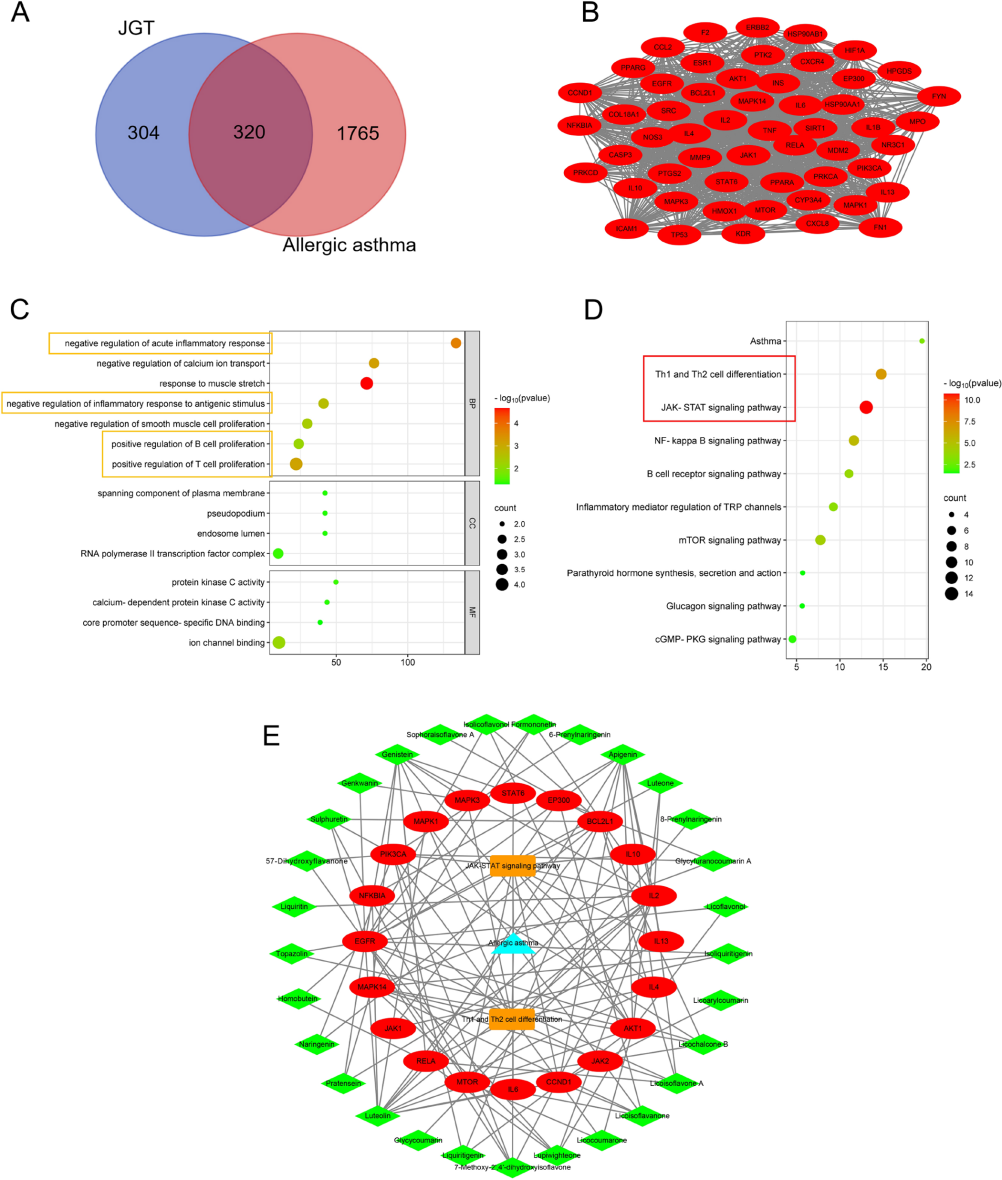
Potential mechanisms of JGT in the treatment of allergic asthma. (A) Venn map: common targets of JGT and allergic asthma. (B) Core targets of JGT in allergic asthma treatment. (C) Biological process of core target enrichment. Yellow boxes represent immune and inflammatory biological processes. (D) Potential pathways for core target enrichment. The red box represents the likely potential pathway. (E) Compound–target–pathways. The green diamond represents the composition, the red ellipse represents the target point, the orange rectangles represent potential pathways, and blue triangles represent diseases.

We employed network pharmacology to investigate how JGT alleviates allergic asthma through its pharmacological mechanisms. Using LC-MS, we identified the main components in JGT, which were associated with 624 targets found in the TCMSP, Swisstarget, and BATMAN databases. Additionally, we found 2085 targets related to allergic asthma in the Genecards and CTD databases. By identifying common targets shared between JGT and allergic asthma, we pinpointed a total of 320 potential targets for treating allergic asthma with JGT (Figure 4A). Subsequently, we created a protein–protein interaction (PPI) network of these potential therapeutic targets using the STRING database. This network was then visualized using Cytoscape 3.2.1, and we calculated the key network parameters. To pinpoint the targets for JGT treatment of allergic asthma with greater precision, we identified 54 core targets. This selection was made using methods that considered topological parameters such as betweenness centrality, closeness centrality, and degree exceeding the average values (Figure 4B). Finally, the DAVID database was used to annotate GO functions and analyze the KEGG pathways associated with these core targets (Figures 4C,D). Network pharmacology revealed that the primary biological process by which JGT improved allergic asthma was the regulation of inflammation and immune responses. Key mechanisms of action included Th1 and Th2 cell differentiation and the JAK-STAT signaling pathway. To visually explain the mechanism of action of JGT, we selected the two pathways for visualization of the compound–target–pathways (Figure 4E).

### 3.4 JGT Inhibited Th2 Cell Differentiation

**Figure 5:**
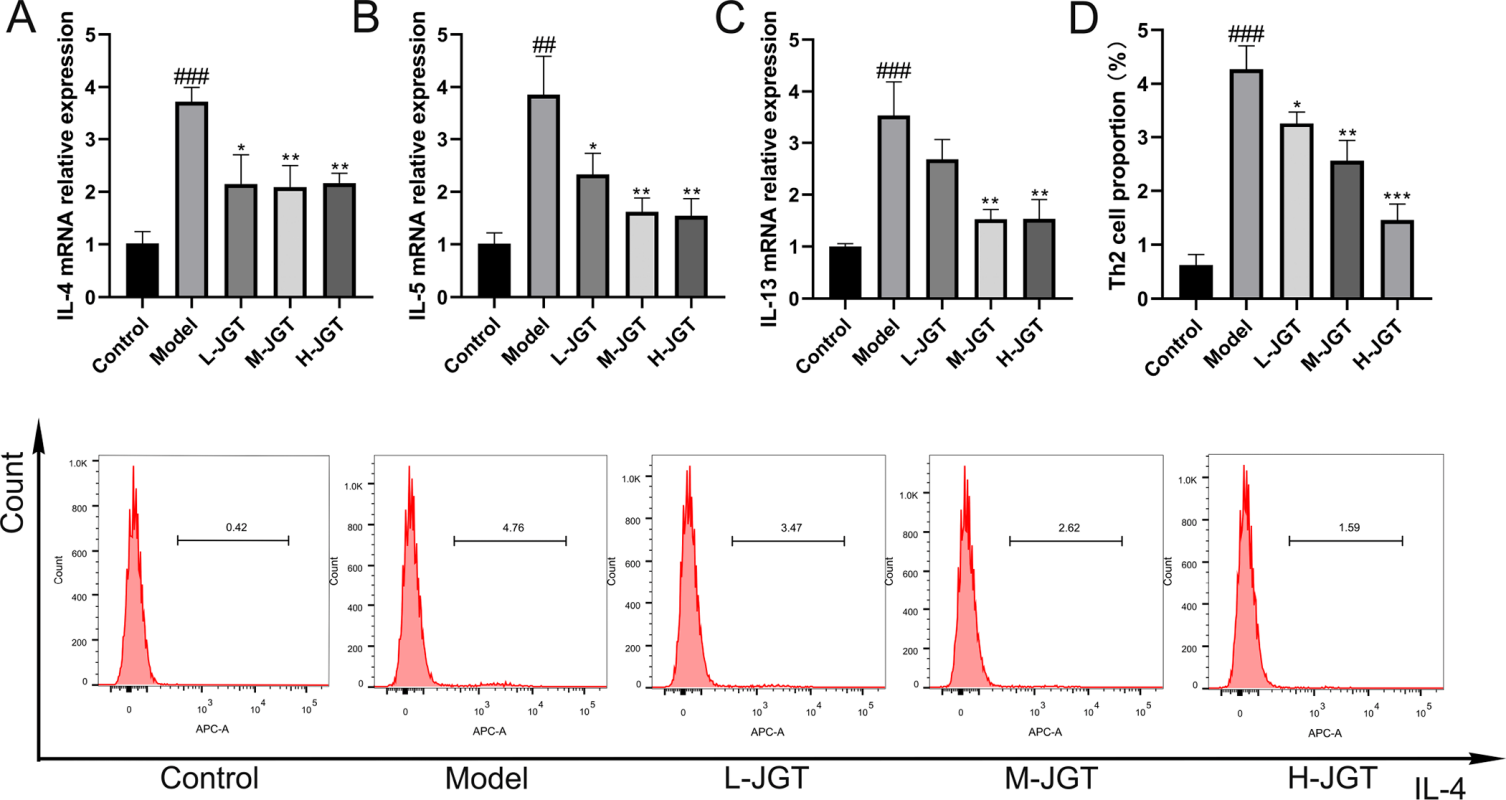
(A–C) Relative mRNA expression levels of IL-4, IL-5, and IL-13 in CD4^+^ T cells (*n* = 3). (D) Proportion of Th2 cells in CD4^+^ T cells (*n* = 3). ^##^*p* < 0.01, ^###^*p* < 0.001, compared to the control group; *p < 0.05, **p < 0.01, ***p < 0.001, compared to the model group.

In this research, our emphasis was on Th2 cell differentiation, given the importance of this subset in allergic asthma due to the secretion of IL-4, IL-5, and IL-13. We utilized qPCR and flow cytometry to examine the impact of JGT on Th2 cell differentiation in CD4^+^ T cells from allergic asthmatic mice. The results revealed substantial upregulation in the mRNA expression of IL-4, IL-5, and IL-13 within the model group in comparison to the control group. Conversely, JGT treatment resulted in a significant decrease in the mRNA expression levels of IL-4, IL-5, and IL-13 (Figures 5A–C). JGT exhibited a dose-dependent reduction in the proportion of Th2 cells among the CD4^+^ T cells (Figure 5D). These results indicated that JGT effectively inhibits the differentiation of Th2 cells.

### 3.5 JGT Suppressed Activation of the JAK1-STAT6 Signaling Pathway in CD4^+^ T Cells

**Figure 6:**
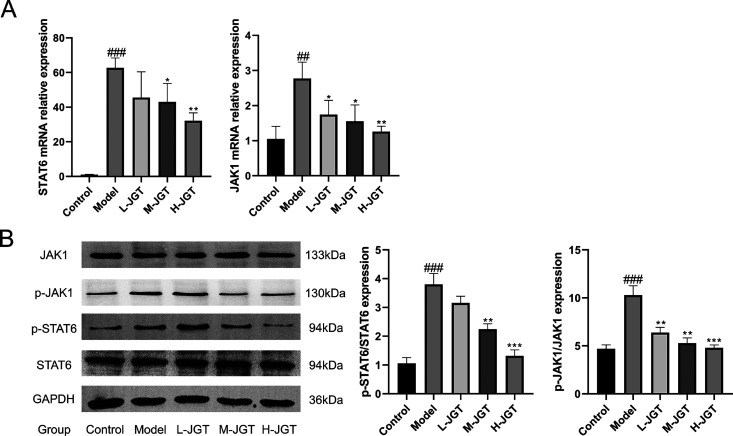
JGT reduced mRNA and protein expression of JAK1 and STAT6 in CD4^+^ T cells. (A) mRNA expression levels of JAK1 and STAT6 within CD4^+^ T cells detected using RT-qPCR (*n* = 3). (B) Protein expression levels of JAK1, STAT6, p-JAK1, and p-STAT6 in CD4^+^ T cells were detected using Western blotting (*n* = 3). ^##^*p* < 0.01, ^###^*p* < 0.001, compared with the control group; *p < 0.05, **p < 0.01, ***p < 0.001, compared with the model group.

To elucidate the mechanism through which JGT inhibits Th2 cell differentiation, we experimentally validated the expression levels of specific target genes and proteins within CD4^+^ T cells, as predicted by network pharmacology. As depicted in Figure 6A, treatment with JGT resulted in a significant reduction in the levels of mRNA expression of JAK1 and STAT6 within CD4^+^ T cells. Furthermore, JGT effectively inhibited the phosphorylation of JAK1 and STAT6 in CD4^+^ T cells, as shown in Figure 6B. These results suggested that JGT could suppress the activation of the JAK1-STAT6 signaling pathway in CD4^+^ T cells.

## 4 Discussion

In this study, we showed how JGT can effectively treat allergic asthma. We used network pharmacological analysis to explore how JGT helps relieve allergic asthma. We used LC-MS to map the chemical composition of JGT and found 38 different compounds. Subsequently, 320 potential targets of JGT ingredients were identified, of which 54 were recognized as key targets. Finally, our analysis indicated that JGT primarily works by affecting inflammation and immune responses, with a focus on Th2 cell differentiation and the JAK-STAT signaling pathway.

In the context of asthma, T helper cells play a crucial role, and among them, Th2 lymphocytes are particularly important in allergic asthma.^9^ This study primarily focuses on the differentiation of Th2 cells given their significant relevance in the context of allergic asthma. Dendritic cells exhibit receptors associated with adaptive immunity, aiding in the internalization of allergens to subsequently present them to CD4^+^ T cell receptors.^10^ Upon activation, CD4^+^ T cells have been demonstrated to undergo differentiation into Th2 cells and release their respective cytokines (IL-4, IL-5, and IL-13). Research indicates a significant increase in Th2 cell cytokine levels in the bronchoalveolar lavage fluid (BALF) of allergic asthma patients and mice.^11^ IL-4 serves as the primary cytokine responsible for initiating the expression of adhesion molecules in B cells, subsequently resulting in the infiltration of eosinophils into inflamed airways.^12^ Meanwhile, IL-5 stands as the principal cytokine accountable for stimulating eosinophil proliferation both in the bloodstream and tissues.^13^ Additionally, IL-13 functions as a critical regulatory factor in bronchial hyper-responsiveness.^14^ In our study, it was evident that the model group showed a marked elevation in the mRNA levels of IL-4, IL-5, and IL-13 within CD4^+^ T cells, along with an increase in the proportion of Th2 cells when compared to the control group. Importantly, JGT can reduce the mRNA levels of IL-4, IL-5, and IL-13 in CD4^+^ T cells along with the proportion of Th2 cells. This suggested that JGT has an inhibitory effect on Th2 cell differentiation.

The JAK/STAT family of proteins plays a pivotal role in immune-mediated disorders, including allergic asthma.^15^ In the context of allergic asthma, a majority of inflammatory pathways are closely associated with cytokines that transmit signals through receptors linked to Janus kinase 1 (JAK1). Signaling cascades dependent on JAK1 play a pivotal role in the pathophysiology of allergic asthma, rendering them attractive targets for therapeutic intervention.^16^ JAK1 phosphorylates and activates the intracellular signal transducer and activator of transcription 6 (STAT6), thereby orchestrating the transcription of downstream target genes.^17^ Several cytokines connected with type 2 inflammation, including IL-4 and IL-13, communicate signals through the JAK-STAT pathway.^18^ In this study, the activation of JAK1 and the subsequent activation of STAT6 were induced by IL-4 and IL-13. The IL-4/IL-13/STAT6 pathway triggers the expression of inflammatory chemokines and holds a central position in the pathophysiology of allergic asthma, particularly with regard to bronchial hyper-responsiveness.^19^ Validation results revealed that phosphorylation levels of JAK1 and STAT6 were higher in CD4^+^ T cells of the model group than in the control group. However, the application of JGT demonstrated a significant reduction in these levels, suggesting the modulation of the JAK1-STAT6 signaling pathway within CD4^+^ T cells through the JGT intervention.

**Figure 7:**
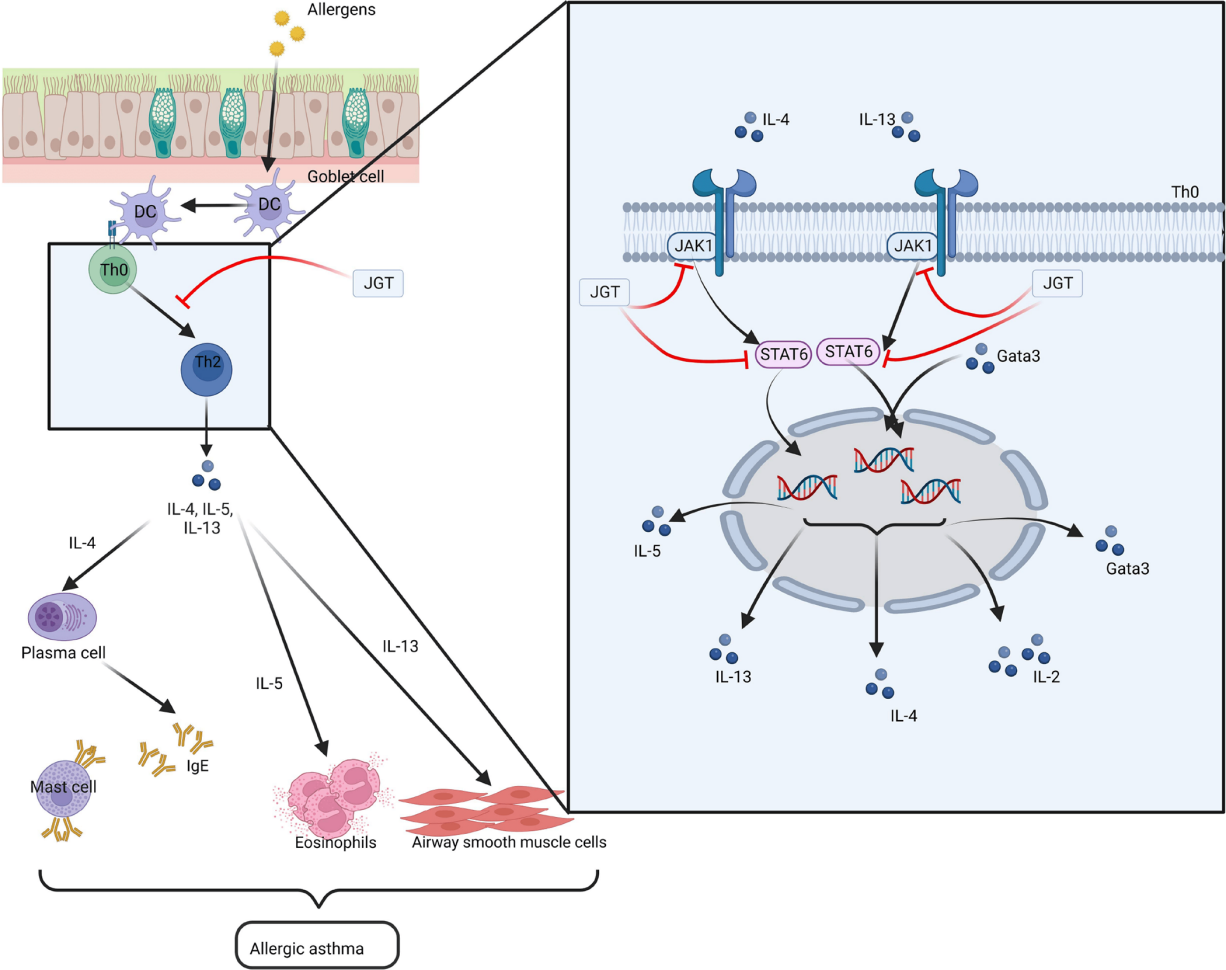
Mechanism of action of JGT in treating allergic asthma. Allergens trigger dendritic cell (DC) activation, prompting their migration to the draining lymph nodes, where they facilitate the differentiation of CD4^+^ T cells from T helper type 0 (Th0) to T helper type 2 (Th2) cells. This shift in the T cell profile results in the production of substantial amounts of Th2 cytokines (IL-4, IL-5, and IL-13), contributing to airway inflammation and bronchial hyper-responsiveness (BHR). Allergic asthma is triggered by two pathological states. The red T-shaped line represents the target of the JGT inhibition.

IL-4 is dependent on the JAK1-STAT6 signal pathway to trigger Th2 cell differentiation, which is the primary pathway implicated in the development of allergic asthma.^20^ When IL-4 binds to the cytokine receptor, JAK1 proteins become activated, leading to the phosphorylation of STAT6, which subsequently forms dimers and translocates to the nucleus.^21^ Experimental validation confirmed that JGT inhibits the differentiation of Th2 cells by blocking the JAK1-STAT6 signaling pathway in CD4^+^ T cells. The associated detailed mechanism of action is depicted in Figure 7.

This study innovatively explores how JGT can effectively manage allergic asthma and uncovers the mechanisms behind its operation. By elucidating the therapeutic potential of this TCM, this study offers a new perspective on managing allergic asthma. Despite these significant findings, additional research exploring alternative pathways is warranted to improve our understanding of the complex mechanisms by which JGT alleviates allergic asthma.

## 5 Conclusions

This study revealed that the administration of JGT ameliorated allergic asthma by impeding the differentiation of Th2 cells through the JAK1-STAT6 signaling pathway. This novel finding is expected to broaden clinicians’ therapeutic choices for managing patients who have been diagnosed with allergic asthma.

## Data Availability

After the publication of the article, the data used in the study results can be obtained by contacting the author.

## Supporting Information Available

(Figure S1) Development of a mouse model of allergic asthma and treatment; (Figure S2) total ion chromatogram obtained in the negative ionization mode; (Figure S3) JGT alleviated airway inflammation in allergic asthma; (Figure S4) potential mechanisms of JGT in the treatment of allergic asthma; (Figure S5) JGT inhibited Th2 cell differentiation; (Figure S6) JGT reduced mRNA and protein expression of JAK1 and STAT6 in CD4^+^ T cells; (Figure S7) language editing certificate; (Table S1) compounds of JGT identified using LC-MS; (Table S2) targets of JGT; (Table S3) targets of allergic asthma; (Table S4) core targets of JGT in allergic asthma treatment (PDF)

## Notes

The animal study described in this research has been subjected to review and approval by the Animal Ethics Committee of Shenzhen Research Institute, Peking University School of Chinese Medicine, with the approval number SZI-AE-2023030101.

## Notes

Z.W. was actively involved in various aspects, encompassing the design of methodology, data curation, drafting of the original manuscript, software development, and formal analysis. Z.L.’s substantial involvement in review and editing ensures the quality and coherence of the paper. Validation tasks were undertaken by W.S., which likely encompass validation of experimental results to ensure the accuracy and reliability of the research. Q.D. was responsible for key tasks such as conceptualization, funding acquisition, review, editing, and visualization. Y.S. oversaw funding acquisition and project management, ensuring the project’s orderly progression.

## Abbreviations List

BALF: bronchoalveolar lavage fluid
BHR: bronchial hyper-responsiveness
DC: dendritic cell
EOS: eosinophil
GO: Gene Ontology
IgE: immunoglobulin E
IL: interleukin
JAK1: Janus kinase 1
JGT: Jiegeng decoction
KEGG: Kyoto Encyclopedia of Genes and Genomes
LC-MS: liquid chromatography–mass spectrometry
OVA: ovalbumin
PPI: protein–protein interaction
STAT6: signal transducer and activator of transcription 6
TCM: traditional Chinese medicine
Th2: T helper type 2
