# Supplementary material for: Integrating Network Pharmacology and Experimental Validation to Elucidate the Mechanism of Jiegeng Decoction in Improving Allergic Asthma
Source: ACS Omega. 2023 Dec 6;8(50):48081–90. doi: 10.1021/acsomega.3c06914 (PMC10733997; doi:10.1021/acsomega.3c06914)
Supplement: Supplementary file 1 — ao3c06914_si_001.pdf [file ao3c06914_si_001.pdf]

Supporting information

Integrating network pharmacology and experimental validation to elucidate the mechanism of Jiegeng decoction in improving allergic asthma

Zhihai Wu[a], *Zhiqiang Luo*[b,c], *Wen Sun*[a], *Yuanyuan Shi*[a,d] **, *Qi Ding*[d] *

* Correspondence Author

** Correspondence Author

[a] School of Life Sciences, Beijing University of Chinese Medicine, Beijing, 100029, China

[b] National Key Laboratory for Quality Ensurance and Sustainable Use of Dao-di Herbs, Beijing 100700, China

[c] State Key Laboratory of Dao-di Herbs, National Resource Center for Chinese Materia Medica, China Academy of Chinese Medical Sciences, Beijing 100700, China

[d] Shenzhen Research Institute, Beijing University of Chinese Medicine, Shenzhen, 518118, China

Figure S1

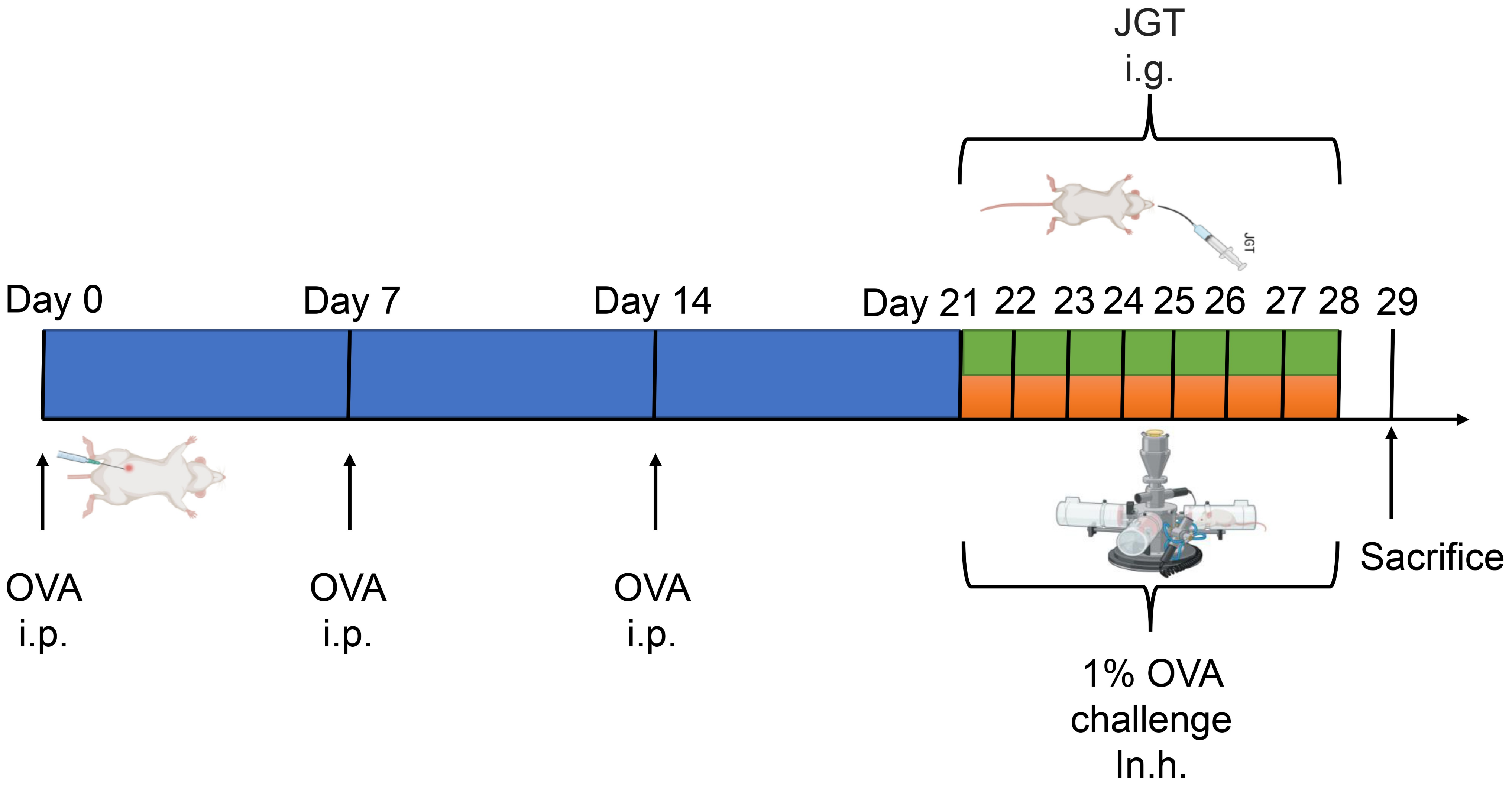

JGT
i.g.
JGT
Day 0
Day 7
Day 14
Day 21
22
23
24
25
26
27
28
29
OVA
i.p.
OVA
i.p.
OVA
i.p.
Sacrifice
1% OVA
challenge
In.h.

Figure S2

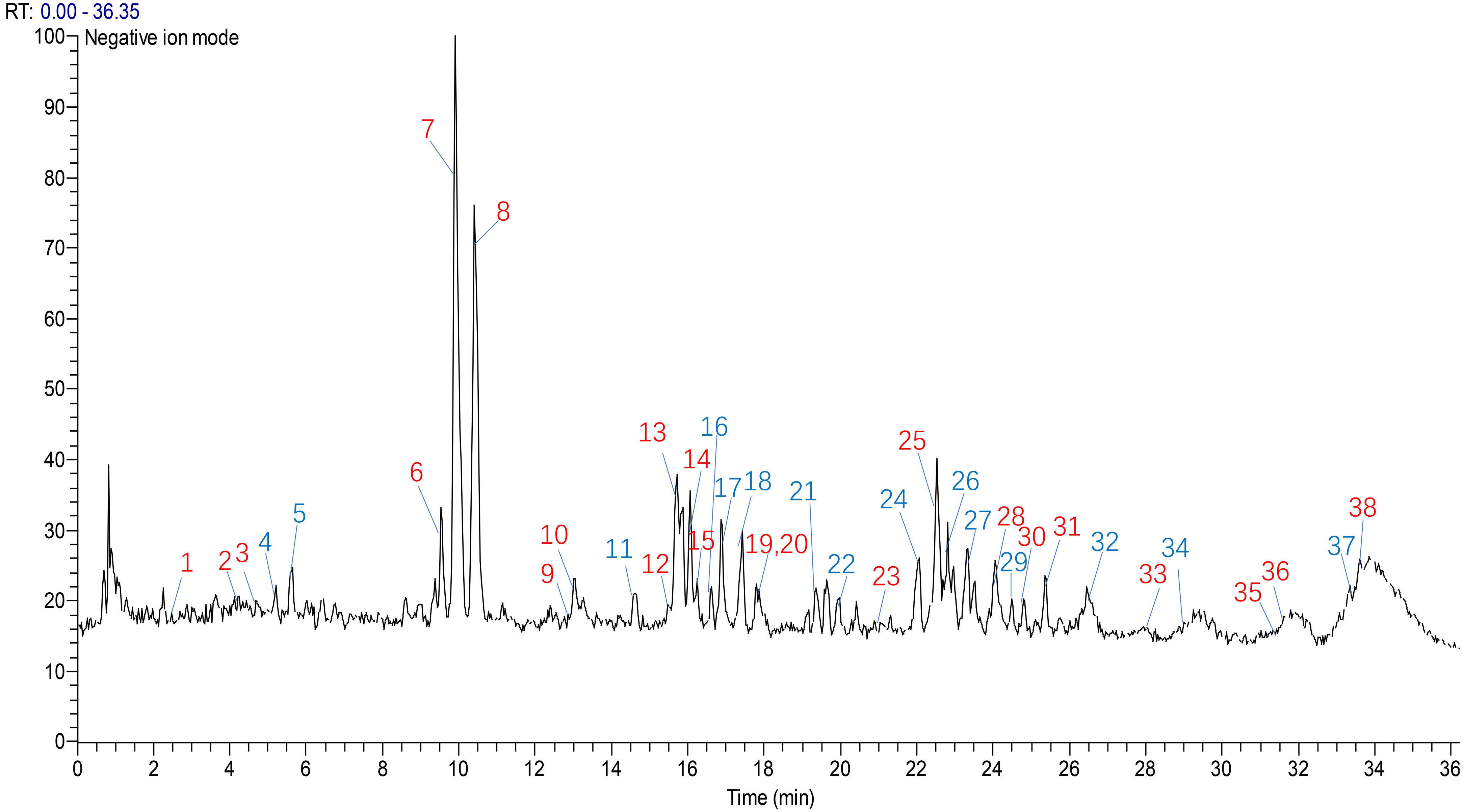
RT: 0.00 - 36.35
Negative ion mode
Time (min)
1
2
3
4
5
6
7
8
9
10
11
12
13
14
15
16
17
18
19,20
21
22
23
24
25
26
27
28
29
30
31
32
33
34
35
36
37
38

Figure S3

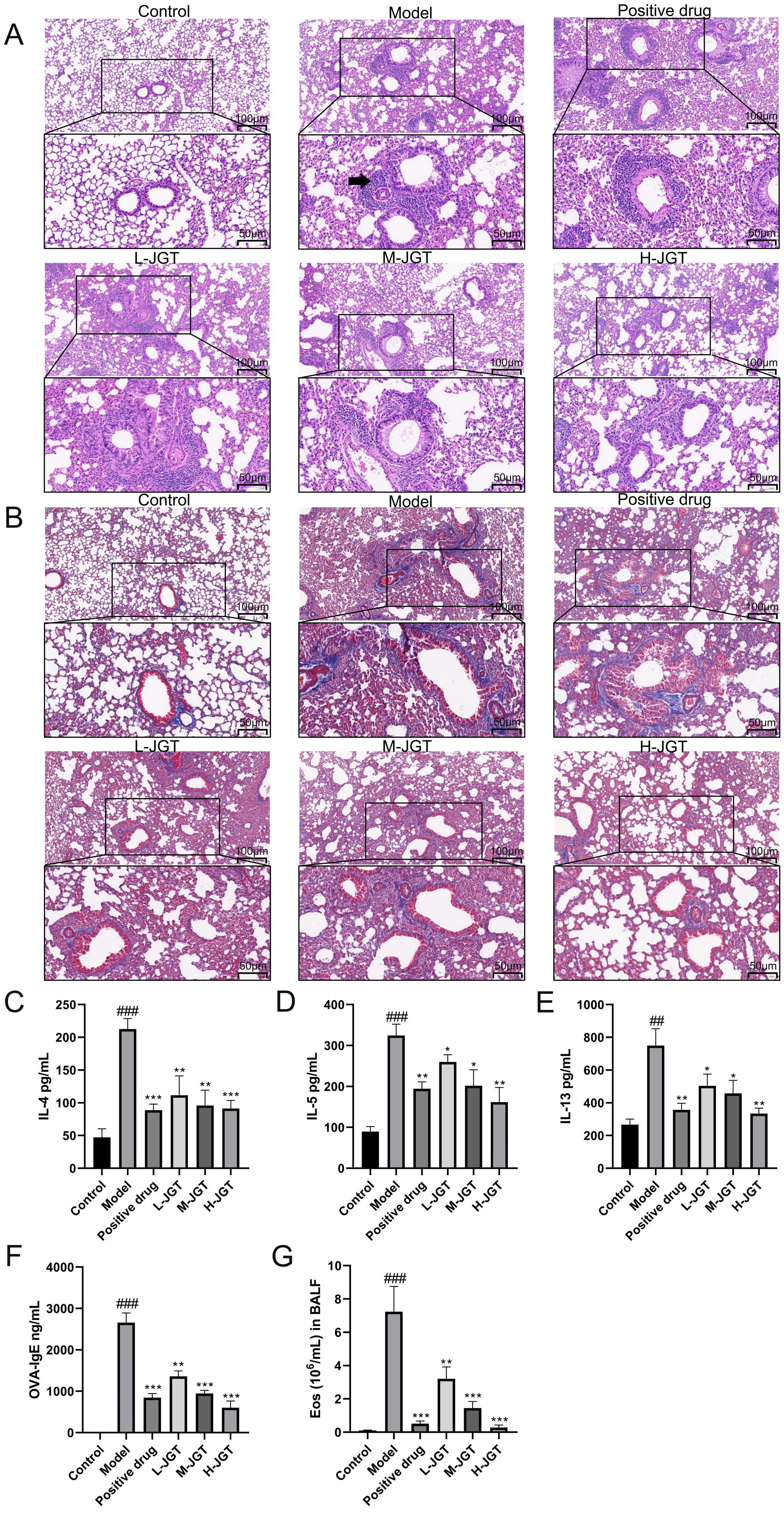
A
Control
Model
Positive drug
100μm
50μm
L-JGT
M-JGT
H-JGT
B
C
IL-4 pg/mL
###
D
IL-5 pg/mL
E
IL-13 pg/mL
##
F
OVA-IgE ng/mL
G
Eos (10^6/mL) in BALF
Control
Model
Positive drug
L-JGT
M-JGT
H-JGT

Figure S4

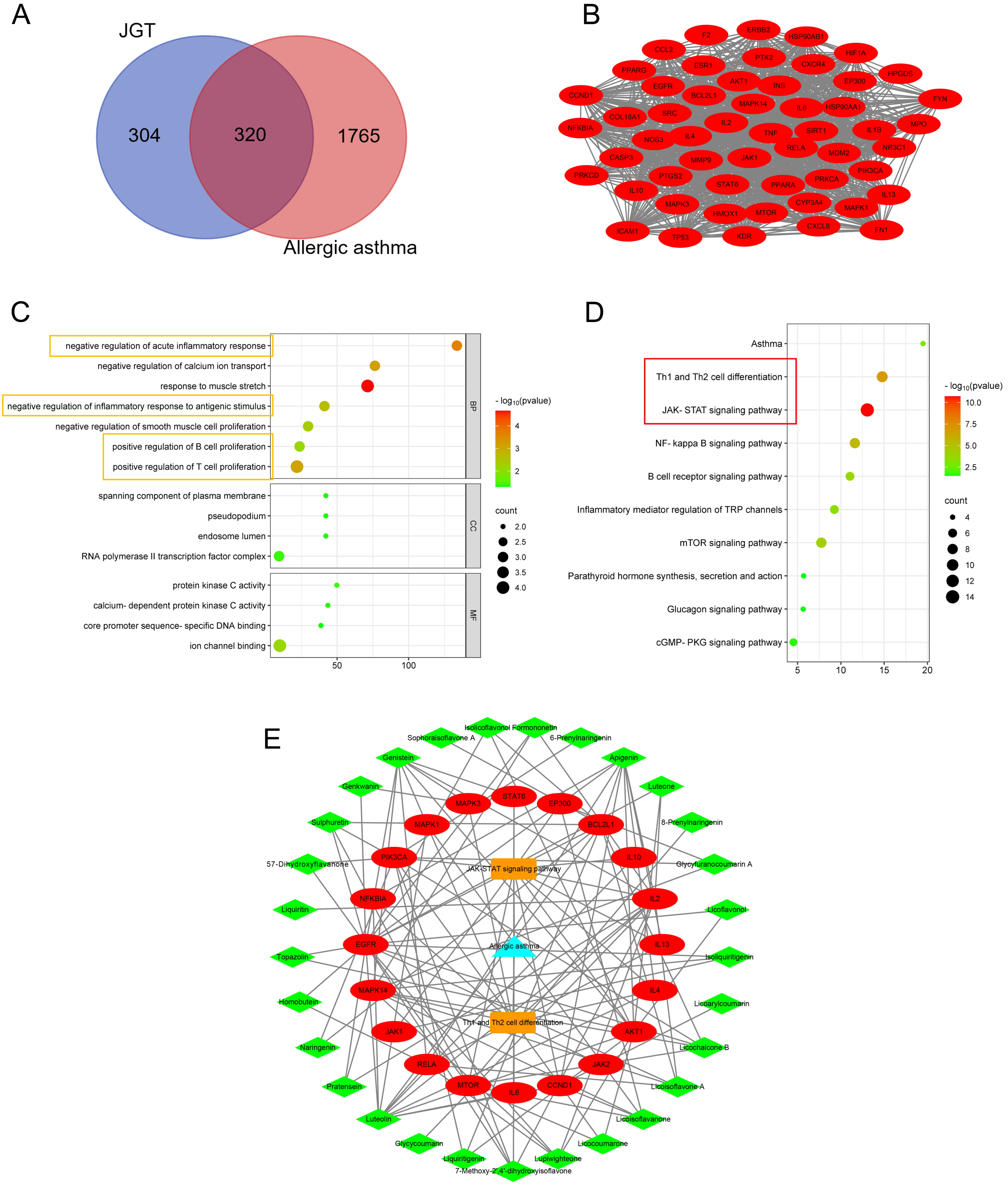

A
JGT
304
320
1765
Allergic asthma
B
F2
ERBB2
HSP90AB1
CCL2
PTK2
HIF1A
PPARG
ESR1
CXCR4
HPGDS
EGFR
AKT1
INS
EP300
CCND1
BCL2L1
FYN
MAPK14
IL6
HSP90AA1
SRC
COL18A1
IL2
MPO
NFKBIA
TNF
SIRT1
IL1B
NOS3
IL4
RELA
NR3C1
MDM2
CASP3
MMP9
JAK1
PIK3CA
PRKCD
PTGS2
STAT6
PPARA
PRKCA
IL10
IL13
MAPK3
CYP3A4
MAPK1
HMOX1
MTOR
CXCL8
ICAM1
FN1
TP53
KDR
C
negative regulation of acute inflammatory response
negative regulation of calcium ion transport
response to muscle stretch
negative regulation of inflammatory response to antigenic stimulus
negative regulation of smooth muscle cell proliferation
positive regulation of B cell proliferation
positive regulation of T cell proliferation
spanning component of plasma membrane
pseudopodium
endosome lumen
RNA polymerase II transcription factor complex
protein kinase C activity
calcium- dependent protein kinase C activity
core promoter sequence- specific DNA binding
ion channel binding
BP
CC
MF
50
100
- log10(pvalue)
4
3
2
count
2.0
2.5
3.0
3.5
4.0
D
Asthma
Th1 and Th2 cell differentiation
JAK- STAT signaling pathway
NF- kappa B signaling pathway
B cell receptor signaling pathway
Inflammatory mediator regulation of TRP channels
mTOR signaling pathway
Parathyroid hormone synthesis, secretion and action
Glucagon signaling pathway
cGMP- PKG signaling pathway
5
10
15
20
- log10(pvalue)
10.0
7.5
5.0
2.5
count
4
6
8
10
12
14
E
Isolicoflavonol
Formononetin
Sophoraisoflavone A
6-Prenylnaringenin
Genistein
Apigenin
Genkwanin
Luteone
Sulphuretin
8-Prenylnaringenin
57-Dihydroxyflavanone
Glycyfuranocoumarin A
Liquiritin
Licoflavonol
Topazolin
Isoliquiritigenin
Homobutein
Licoarylcoumarin
Naringenin
Licochalcone B
Pratensein
Licoisoflavone A
Luteolin
Licoisoflavanone
Glycycoumarin
Licocoumarone
Liquiritigenin
Lupiwighteone
7-Methoxy-2',4'-dihydroxyisoflavone
JAK-STAT signaling pathway
Allergic asthma
Th1 and Th2 cell differentiation
MAPK3
STAT6
EP300
MAPK1
BCL2L1
PIK3CA
IL10
NFKBIA
IL2
EGFR
IL13
MAPK14
IL4
JAK1
AKT1
RELA
JAK2
MTOR
IL6
CCND1

Figure S5

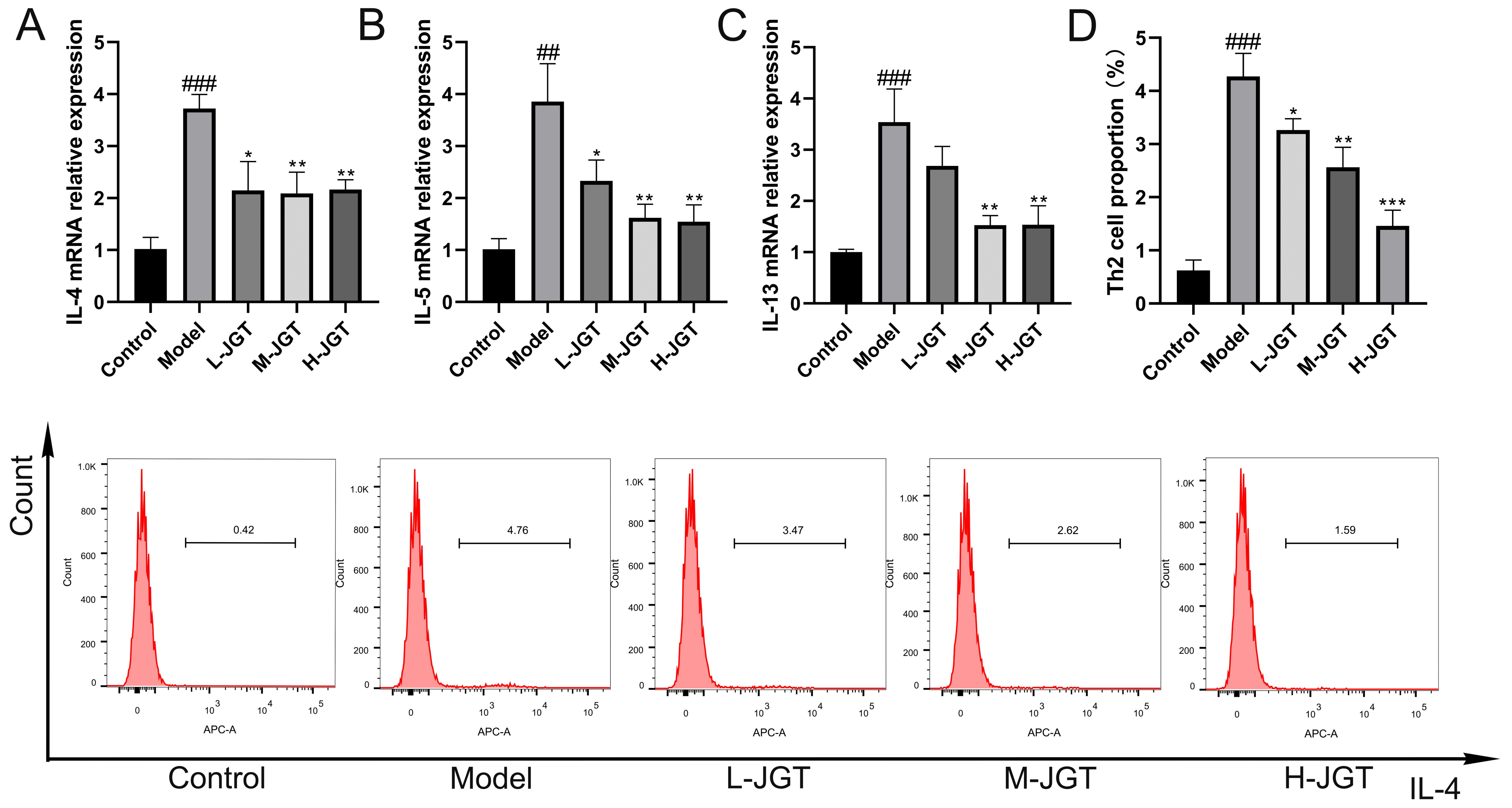
A
IL-4 mRNA relative expression
B
IL-5 mRNA relative expression
C
IL-13 mRNA relative expression
D
Th2 cell proportion（%）
###
##
###
###
Control
Model
L-JGT
M-JGT
H-JGT
Count
APC-A
0.42
4.76
3.47
2.62
1.59
IL-4

Figure S6

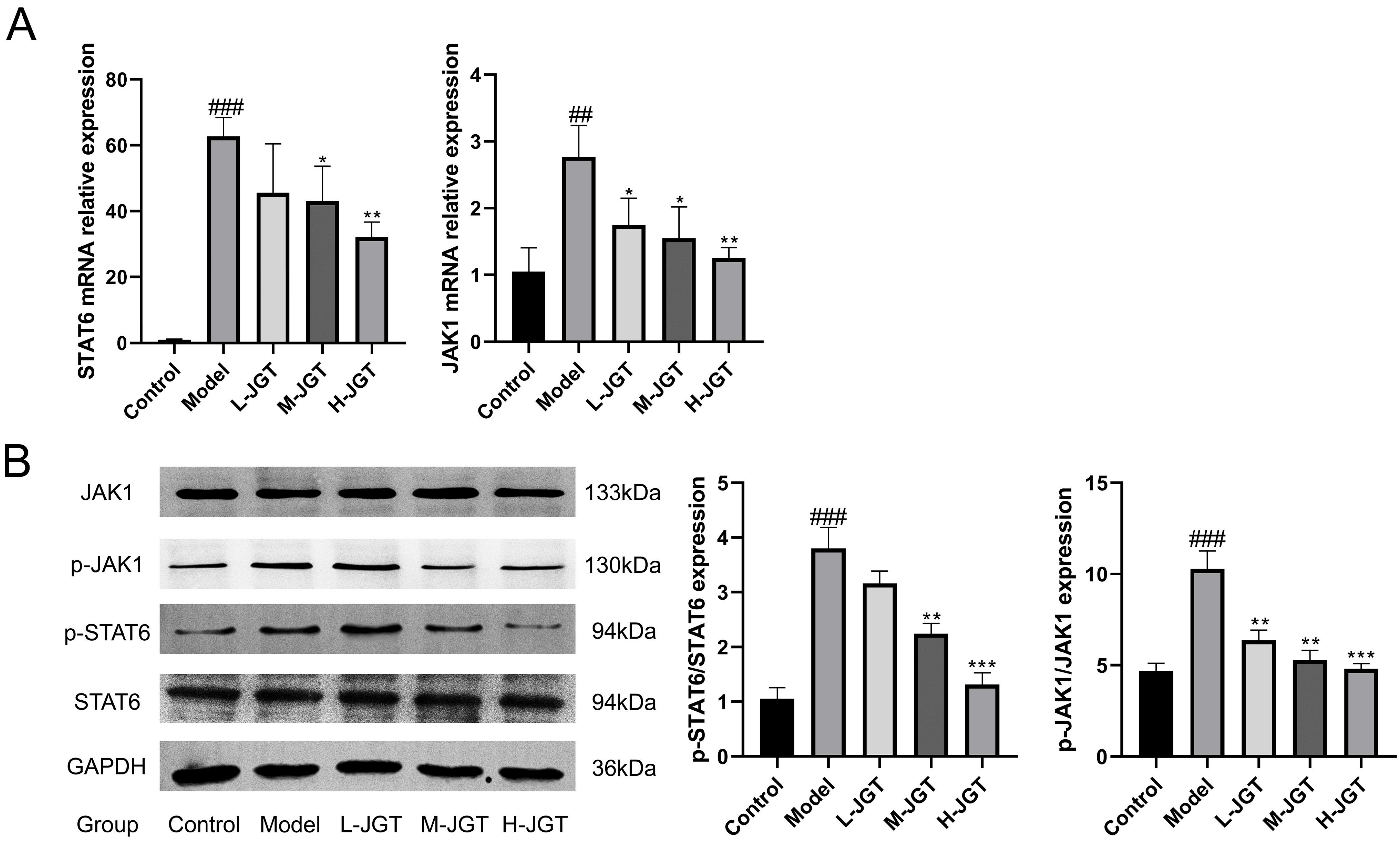
A
STAT6 mRNA relative expression
0
20
40
60
80
###
*
**
Control
Model
L-JGT
M-JGT
H-JGT
JAK1 mRNA relative expression
0
1
2
3
4
##
*
*
**
B
JAK1
p-JAK1
p-STAT6
STAT6
GAPDH
133kDa
130kDa
94kDa
94kDa
36kDa
Group
Control
Model
L-JGT
M-JGT
H-JGT
p-STAT6/STAT6 expression
5
###
**
***
p-JAK1/JAK1 expression
10
15
***

Table S1. Compounds of JGT identified using LC-MS.

No	Identification	Formula	RT (min)	Quasi-molecular ion	*m/z*	Fragments(*m/z*)	Error (ppm)
1	Isoliquiritigenin[1]	$C_{15}H_{12}O_4$	2.66	$[M-H]^-$	255.0651	165.0669, 86.9909	-1.52
2	Liquiritin[2]	$C_{21}H_{22}O_9$	4.06	$[M-H]^-$	417.1180	89.0243, 194.0580, 176.0477, 161.0244	-1.24
3	Isolicoflavonol[3]	$C_{20}H_{18}O_6$	4.54	$[M-H]^-$	353.1019	173.0454, 179.0355	-0.64
4	Asperulosidic acid[4]	$C_{18}H_{24}O_{12}$	5.22	$[M-H]^-$	432.1262	193.0354, 376.5678	-0.28
5	Morinlongoside A[5]	$C_{29}H_{38}O_{15}$	5.63	$[M-H]^-$	625.2127	497.1129, 205.0718	-0.67
6	Neoliquiritin[6]	$C_{21}H_{22}O_9$	9.85	$[M-H]^-$	417.1180	153.0193, 154.0228	-1.63
7	Liquiritigenin[7]	$C_{15}H_{12}O_4$	9.89	$[M-H]^-$	255.0651	119.0501, 135.0087	-2.32
8	Liquiritin apioside[8]	$C_{26}H_{30}O_{13}$	10.39	$[M-H]^-$	549.1613	255.0663, 135.0087	-0.54
9	Pratensein[9]	$C_{16}H_{12}O_6$	12.89	$[M-H]^-$	299.0550	299.0562, 199.04005	-0.42
10	Genistein[10]	$C_{15}H_{10}O_5$	13.06	$[M-H]^-$	269.0444	269.0457, 133.0294	-1.84
11	Lusitanicoside[11]	$C_{21}H_{30}O_{10}$	14.62	$[M-H]^-$	442.1833	353.0668, 383.0775, 325.0712	-1.95
12	Glycycoumarin[12]	$C_{21}H_{20}O_6$	15.53	$[M-H]^-$	367.1176	112.985, 68.9957	-2.41
13	Formononetin[13]	$C_{16}H_{12}O_4$	15.63	$[M-H]^-$	267.0651	252.0430, 267.0663	-2.15
14	Licochalcone B[14]	$C_{16}H_{14}O_5$	16.11	$[M-H]^-$	285.0757	150.0321, 270.0534	-0.32
15	Luteolin[15]	$C_{15}H_{10}O_5$	16.24	$[M-H]^-$	269.0444	177.0193, 151.0033	-0.72
16	Licuroside[16]	$C_{26}H_{30}O_{13}$	16.62	$[M-H]^-$	549.1611	119.0501, 153.0193, 297.0760, 91.0189	-2.14
17	Platycodon A[17]	$C_{42}H_{68}O_{16}$	16.94	$[M-H]^-$	827.4423	483.1744, 707.4003, 681.3784	-1.36
18	Uralsaponin F[18]	$C_{44}H_{64}O_{19}$	17.01	$[M-H]^-$	895.3958	827.4434, 665.3915, 255.0663, 531.1493	-0.97
19	Genkwanin[19]	$C_{16}H_{12}O_5$	17.85	$[M-H]^-$	283.0601	268.0378, 152.5543	-1.39

20	7-Methoxy-2',4'-dihydroxyisoflavone[20]	$C_{16}H_{12}O_5$	17.85	$[M-H]^-$	283.0601	150.03227 270.05353	-1.05
21	Platyconic acid E[21]	$C_{58}H_{92}O_{30}$	19.38	$[M-H]^-$	1267.5589	783.4173, 485.2910, 557.3125, 807.7927	-2.48
22	Naringenin[22]	$C_{15}H_{12}O_5$	19.9	$[M-H]^-$	271.0601	151.0036, 119.0502	-1.63
23	Apigenin[23]	$C_{15}H_{10}O_5$	21.01	$[M-H]^-$	269.0444	108.0217, 133.0294, 117.0341	-0.98
24	Platycodin J[24]	$C_{57}H_{90}O_{29}$	22.09	$[M-H]^-$	1237.5484	1219.5466, 695.3655, 633.3647, 519.3329	-0.45
25	Polygalacin D2[25]	$C_{63}H_{102}O_{32}$	22.65	$[M-H]^-$	1369.6270	469.1561, 503.3379, 647.3803, 827.4440	-0.08
26	Platyconic acid C[26]	$C_{52}H_{82}O_{25}$	22.79	$[M-H]^-$	1105.5061	1075.4957, 895.4345, 974.8721, 665.3538	-1.68
27	Platyconic acid A[27]	$C_{57}H_{90}O_{29}$	23.21	$[M-H]^-$	1237.5484	937.4340, 1207.5382, 1027.4760, 485.2912	-2.16
28	Platycodin C[28]	$C_{59}H_{94}O_{29}$	23.97	$[M-H]^-$	1265.5797	1133.5470, 681.3860, 723.3963, 469.1562	-1.35
29	5,7-Dihydroxyflavanone[29]	$C_{15}H_{12}O_4$	24.39	$[M-H]^-$	255.0651	119.0502, 135.0088	-0.45
30	Glycyrrhizin[30]	$C_{42}H_{62}O_{16}$	24.99	$[M-H]^-$	821.3954	512.1426, 424.2771, 516.8211, 509.0119	-0.39
31	Uralsaponin B[31]	$C_{42}H_{62}O_{16}$	25.4	$[M-H]^-$	821.3954	645.3633, 544.7770, 517.7642, 438.4532	-2.15
32	8-Prenylnaringenin[32]	$C_{20}H_{20}O_5$	26.89	$[M-H]^-$	339.1227	135.0451, 203.0714, 221.0819	-2.01
33	Licocoumarone[33]	$C_{20}H_{20}O_5$	28.08	$[M-H]^-$	339.1227	109.0295, 163.0036	-0.14
34	Topazolin[34]	$C_{21}H_{20}O_6$	29.08	$[M-H]^-$	367.1176	309.0407, 367.1187	-2.04
35	Luteone[35]	$C_{20}H_{18}0_6$	31.45	$[M-H]^-$	353.1019	125.0244, 227.0715	-0.24
36	Licoisoflavone A[36]	$C_{20}H_{18}0_6$	31.63	$[M-H]^-$	353.1019	150.0322, 177.0193	-0.68
37	Glycyfuranocoumarin A[37]	$C_{21}H_{20}O_6$	33.5	$[M-H]^-$	367.1176	309.0407, 284.1189	-0.46
38	Lupiwighteone[38]	$C_{21}H_{20}O_6$	33.7	$[M-H]^-$	367.1176	349.0406, 246.1189	-0.12

Reference

(1) Chen, T.; Deng, S.; Lin, R. The inhibitory effect of Isoliquiritigenin on the proliferation of human arterial smooth muscle cell. *BMC pharmacology & toxicology* **2017**, *18* (1), 57. DOI: 10.1186/s40360-017-0165-2 From NLM.

(2) Chen, Y.; Tao, B.; Deng, X.; Wang, X.; Zhang, M.; Cao, Y.; Wei, Z.; Sun, S. A novel electrochemical sensor based on N, S co-doped liquorice carbon/functionalized MWCNTs nanocomposites for simultaneous detection of licochalcone A and liquiritin. *Talanta* **2023**, *252*, 123869. DOI: 10.1016/j.talanta.2022.123869 From NLM.

(3) Han, L.; Yuan, Y.; Zhao, L.; He, Q.; Li, Y.; Chen, X.; Liu, X.; Liu, K. Tracking antiangiogenic components from Glycyrrhiza uralensis Fisch. based on zebrafish assays using high-speed countercurrent chromatography. *Journal of separation science* **2012**, *35* (9), 1167-1172. DOI: 10.1002/jssc.201101031 From NLM.

(4) Deng, S.; West, B. J.; Palu, K.; Jensen, C. J. Determination and comparative analysis of major iridoids in different parts and cultivation sources of Morinda citrifolia. *Phytochemical analysis : PCA* **2011**, *22* (1), 26-30. DOI: 10.1002/pca.1246 From NLM.

(5) Cuong, N. M.; Huong, T. T.; Son, N. T.; Cuong, T. D.; Van, D. T.; Khanh, P. N.; Ha, V. T.; Tram, N. C.; Long, P. Q.; Kim, Y. H. Morinlongosides A-C, Two New Naphthalene Glycoside and a New Iridoid Glycoside from the Roots of Morinda longissima. *Chemical & pharmaceutical bulletin* **2016**, *64* (8), 1230-1234. DOI: 10.1248/cpb.c15-01039 From NLM.

(6) Bai, H.; Bao, F.; Fan, X.; Han, S.; Zheng, W.; Sun, L.; Yan, N.; Du, H.; Zhao, H.; Yang, Z. Metabolomics study of different parts of licorice from different geographical origins and their anti-inflammatory activities. *Journal of separation science* **2020**, *43* (8), 1593-1602. DOI: 10.1002/jssc.201901013 From NLM.

(7) Sayre, C. L.; Hopkins, M.; Takemoto, J. K.; Davies, N. M. Chiral analytical method development of liquiritigenin with application to a pharmacokinetic study. *Biomedical chromatography : BMC* **2013**, *27* (3), 404-406. DOI: 10.1002/bmc.2787 From NLM.

(8) Guan, Y.; Li, F. F.; Hong, L.; Yan, X. F.; Tan, G. L.; He, J. S.; Dong, X. W.; Bao, M. J.; Xie, Q. M. Protective effects of liquiritin apioside on cigarette smoke-induced lung epithelial cell injury. *Fundamental & clinical pharmacology* **2012**, *26* (4), 473-483. DOI: 10.1111/j.1472-8206.2011.00956.x From NLM.

(9) Liang, C.; Tan, S.; Huang, Q.; Lin, J.; Lu, Z.; Lin, X. Pratensein ameliorates β-amyloid-induced cognitive impairment in rats via reducing oxidative damage and restoring synapse and BDNF levels. *Neuroscience letters* **2015**, *592*, 48-53. DOI: 10.1016/j.neulet.2015.03.003 From NLM.

(10) Multigenerational reproductive study of genistein (Cas No. 446-72-0) in Sprague-Dawley rats (feed study). *National Toxicology Program technical report series* **2008**, (539), 1-266. From NLM.

(11) Abrams, R. P. M.; Yasgar, A.; Teramoto, T.; Lee, M. H.; Dorjsuren, D.; Eastman, R. T.; Malik, N.; Zakharov, A. V.; Li, W.; Bachani, M.; et al. Therapeutic candidates for the Zika virus identified by a high-throughput screen for Zika protease inhibitors. *Proceedings of the National Academy of Sciences of the United States of America* **2020**, *117* (49), 31365-31375. DOI: 10.1073/pnas.2005463117 From NLM.

(12) Wang, Q.; Qiao, X.; Liu, C. F.; Ji, S.; Feng, L. M.; Qian, Y.; Guo, D. A.; Ye, M. Metabolites identification of glycycoumarin, a major bioactive coumarin from licorice in rats. *Journal of pharmaceutical and biomedical analysis* **2014**, *98*, 287-295. DOI: 10.1016/j.jpba.2014.06.001 From NLM.

(13) Park, Y.; Choo, S. P.; Jung, G. S.; Kim, S.; Lee, M. J.; Im, W.; Park, H.; Lee, I.; Lee, J. H.; Cho, S.; et al. Formononetin Inhibits Progression of Endometriosis via Regulation of p27, pSTAT3, and Progesterone Receptor: In Vitro and In Vivo Studies. *Nutrients* **2023**, *15* (13). DOI: 10.3390/nu15133001 From NLM.

(14) Furusawa, J.; Funakoshi-Tago, M.; Mashino, T.; Tago, K.; Inoue, H.; Sonoda, Y.; Kasahara, T. Glycyrrhiza inflata-derived chalcones, Licochalcone A, Licochalcone B and Licochalcone D, inhibit phosphorylation of NF-kappaB p65 in LPS signaling pathway. *International immunopharmacology* **2009**, *9* (4), 499-507. DOI: 10.1016/j.intimp.2009.01.031 From NLM.

(15) Lin, L. C.; Pai, Y. F.; Tsai, T. H. Isolation of Luteolin and Luteolin-7-O-glucoside from Dendranthema morifolium Ramat Tzvel and Their Pharmacokinetics in Rats. *Journal of agricultural and food chemistry* **2015**, *63* (35), 7700-7706. DOI: 10.1021/jf505848z From NLM.

(16) Boyko, N.; Zhilyakova, E.; Malyutina, A.; Novikov, O.; Pisarev, D.; Abramovich, R.; Potanina, O.; Lazar, S.; Mizina, P.; Sahaidak-Nikitiuk, R. Studying and Modeling of the Extraction Properties of the Natural Deep Eutectic Solvent and Sorbitol-Based Solvents in Regard to Biologically Active Substances from Glycyrrhizae Roots. *Molecules (Basel, Switzerland)* **2020**, *25* (7). DOI: 10.3390/molecules25071482 From NLM.

(17) Hwang, K. A.; Hwang, Y. J.; Im, P. R.; Hwang, H. J.; Song, J.; Kim, Y. J. Platycodon grandiflorum Extract Reduces High-Fat Diet-Induced Obesity Through Regulation of Adipogenesis and Lipogenesis Pathways in Mice. *Journal of medicinal food* **2019**, *22* (10), 993-999. DOI: 10.1089/jmf.2018.4370 From NLM.

(18) Cai, L. N.; Zhang, R. Y.; Zhang, Z. L.; Wang, B.; Qiao, L.; Huang, L. R.; Cheng, J. R. [The structure of glyeurysaponin]. *Yao xue xue bao = Acta pharmaceutica Sinica* **1991**, *26* (6), 447-450. From NLM.

(19) Gao, Y.; Liu, F.; Fang, L.; Cai, R.; Zong, C.; Qi, Y. Genkwanin inhibits proinflammatory mediators mainly through the regulation of miR-101/MKP-1/MAPK pathway in LPS-activated macrophages. *PloS one* **2014**, *9* (5), e96741. DOI: 10.1371/journal.pone.0096741 From NLM.

(20) Becker, A.; Yamada, Y.; Sato, F. California poppy (Eschscholzia californica), the Papaveraceae golden girl model organism for evodevo and specialized metabolism. *Frontiers in plant science* **2023**, *14*, 1084358. DOI: 10.3389/fpls.2023.1084358 From NLM.

(21) Li, L. J.; Liu, Z. H.; Chen, Y.; Tian, J. K. [Chemical constituents from roots of Platycodon grandiflorum]. *Zhongguo Zhong yao za zhi = Zhongguo zhongyao zazhi = China journal of Chinese materia medica* **2006**, *31* (18), 1506-1509. From NLM.

(22) Hsiu, S. L.; Huang, T. Y.; Hou, Y. C.; Chin, D. H.; Chao, P. D. Comparison of metabolic pharmacokinetics of naringin and naringenin in rabbits. *Life sciences* **2002**, *70* (13), 1481-1489. DOI: 10.1016/s0024-3205(01)01491-6 From NLM.

(23) Wu, W.; Zu, Y.; Wang, L.; Wang, L.; Wang, H.; Li, Y.; Wu, M.; Zhao, X.; Fu, Y. Preparation, characterization and antitumor activity evaluation of apigenin nanoparticles by the liquid antisolvent precipitation technique. *Drug delivery* **2017**, *24* (1), 1713-1720. DOI: 10.1080/10717544.2017.1399302 From NLM.

(24) Chen, S.; Wang, Q.; Ming, S.; Zheng, H.; Hua, B.; Yang, H. S. Platycodin D induces apoptosis through JNK1/AP-1/PUMA pathway in non-small cell lung cancer cells: A new mechanism for an old compound. *Frontiers in pharmacology* **2022**, *13*, 1045375. DOI: 10.3389/fphar.2022.1045375 From NLM.
(25) Choi, J. H.; Yoo, K. Y.; Park, O. K.; Lee, C. H.; Won, M. H.; Hwang, I. K.; Ryu, S. Y.; Kim, Y. S.; Yi, J. S.; Bae, Y. S.; et al. Platycodin D and 2''-O-acetyl-polygalacin D2 isolated from Platycodon grandiflorum protect ischemia/reperfusion injury in the gerbil hippocampus. *Brain research* **2009**, *1279*, 197-208. DOI: 10.1016/j.brainres.2009.05.005 From NLM.
(26) Ma, X. Q.; Li, S. M.; Chan, C. L.; Su, T.; Li, W. D.; Cao, H.; Fong, W. F.; Yu, Z. L. Influence of sulfur fumigation on glycoside profile in Platycodonis Radix (Jiegeng). *Chinese medicine* **2016**, *11*, 32. DOI: 10.1186/s13020-016-0101-1 From NLM.
(27) Choi, Y. H.; Yoo, D. S.; Choi, C. W.; Cha, M. R.; Kim, Y. S.; Lee, H. S.; Lee, K. R.; Ryu, S. Y. Platyconic acid A, a genuine triterpenoid saponin from the roots of Platycodon grandiflorum. *Molecules (Basel, Switzerland)* **2008**, *13* (11), 2871-2879. DOI: 10.3390/molecules13112871 From NLM.
(28) Choi, Y. H.; Yoo, D. S.; Cha, M. R.; Choi, C. W.; Kim, Y. S.; Choi, S. U.; Lee, K. R.; Ryu, S. Y. Antiproliferative effects of saponins from the roots of Platycodon grandiflorum on cultured human tumor cells. *Journal of natural products* **2010**, *73* (11), 1863-1867. DOI: 10.1021/np100496p From NLM.
(29) Zhang, X.; Khalidi, O.; Kim, S. Y.; Wang, R.; Schultz, V.; Cress, B. F.; Gross, R. A.; Koffas, M. A. G.; Linhardt, R. J. Synthesis and biological evaluation of 5,7-dihydroxyflavanone derivatives as antimicrobial agents. *Bioorganic & medicinal chemistry letters* **2016**, *26* (13), 3089-3092. DOI: 10.1016/j.bmcl.2016.05.003 From NLM.
(30) Jitrangsri, K.; Kamata, K.; Akiba, M.; Yajiri, Y.; Ishibashi, M.; Tatsuzaki, J.; Ishikawa, T. Is 18α-Glycyrrhizin a real natural product? Improved preparation of 18α-Glycyrrhizin from 18β-Glycyrrhizin as a positive standard for HPLC analysis of licorice extracts. *Journal of natural medicines* **2022**, *76* (2), 367-378. DOI: 10.1007/s11418-021-01589-9 From NLM.
(31) Zeng, L.; Zhang, R. Y.; Lou, Z. C. [Separation and quantitative determination of three saponins in licorice root by high performance liquid chromatography]. *Yao xue xue bao = Acta pharmaceutica Sinica* **1991**, *26* (1), 53-58. From NLM.
(32) Diel, P.; Thomae, R. B.; Caldarelli, A.; Zierau, O.; Kolba, S.; Schmidt, S.; Schwab, P.; Metz, P.; Vollmer, G. Regulation of gene expression by 8-prenylnaringenin in uterus and liver of Wistar rats. *Planta medica* **2004**, *70* (1), 39-44. DOI: 10.1055/s-2004-815453 From NLM.
(33) Zhao, C.; Wang, D.; Gao, Z.; Kan, H.; Qiu, F.; Chen, L.; Li, H. Licocoumarone induces BxPC-3 pancreatic adenocarcinoma cell death by inhibiting DYRK1A. *Chemico-biological interactions* **2020**, *316*, 108913. DOI: 10.1016/j.cbi.2019.108913 From NLM.
(34) Kırmızıbekmez, H.; Uysal, G. B.; Masullo, M.; Demirci, F.; Bağcı, Y.; Kan, Y.; Piacente, S. Prenylated polyphenolic compounds from Glycyrrhiza iconica and their antimicrobial and antioxidant activities. *Fitoterapia* **2015**, *103*, 289-293. DOI: 10.1016/j.fitote.2015.05.003 From NLM.
(35) Morkunas, I.; Marczak, Ł.; Stachowiak, J.; Stobiecki, M. Sucrose-induced lupine defense against Fusarium oxysporum. Sucrose-stimulated accumulation of isoflavonoids as a defense response of lupine to Fusarium oxysporum. *Plant physiology and biochemistry : PPB* **2005**, *43* (4), 363-373. DOI: 10.1016/j.plaphy.2005.02.011 From NLM.
(36) Guo, R.; Liu, N.; Liu, H.; Zhang, J.; Zhang, H.; Wang, Y.; Baruscotti, M.; Zhao, L.; Wang, Y. High content screening identifies licoisoflavone A as a bioactive compound of Tongmaiyangxin Pills to restrain cardiomyocyte hypertrophy via activating Sirt3. *Phytomedicine : international journal of phytotherapy and phytopharmacology* **2020**, *68*, 153171. DOI: 10.1016/j.phymed.2020.153171 From NLM.
(37) Blajchman, M. A. Novel platelet products, substitutes and alternatives. *Transfusion clinique et biologique : journal de la Societe francaise de transfusion sanguine* **2001**, *8* (3), 267-271. DOI: 10.1016/s1246-7820(01)00127-6 From NLM.
(38) Won, Y. S.; Seo, K. I. Lupiwighteone induces caspase-dependent and -independent apoptosis on human breast cancer cells via inhibiting PI3K/Akt/mTOR pathway. *Food and chemical toxicology : an international journal published for the British Industrial Biological Research Association* **2020**, *135*, 110863. DOI: 10.1016/j.fct.2019.110863 From NLM.

Table S2. The targets of JGT

BCL2L1	NAALAD2	SOAT1	MTTP	SOAT2	AKR1B1	SRD5A1	TYR	XDH	EPHX2
NMUR2	ADRA2A	ADRA2C	ADORA2A	ADORA3	SLC29A1	CA14	CHIA	SLC5A2	SLC5A1
SLC5A4	ADORA1	ADORA2B	ADK	FUCA1	HRAS	GBA	MMP13	MMP1	MMP7
MMP8	HSPA8	HSPA5	EGFR	CDC25B	MGMT	LGALS7	MAPK14	IGFBP3	TOP1
IMPDH1	MME	ECE1	DHFR	AKR1C3	ABL1	EPHA2	LCK	SRC	KDR
MAP3K9	FGFR1	AURKA	BTK	NADK	AGTR1	EIF4H	PABPC1	ATIC	ALOX12
HK2	HK1	CA4	CA5A	CYP19A1	SLC28A3	EIF4A1	PTGS1	MMP12	MAOB
CYP1B1	TAS2R31	TDP1	ABCC1	HSD17B1	SHBG	CBR1	F2	AR	NR3C1
IL2	STAT3	PPP2CA	GLI1	PTPN1	PTAFR	TLR9	HTR2B	ADRA2B	DRD1
ADRA1D	HTR2A	HTR2C	ADRA1A	DRD3	CYP2D6	HTR6	HTR1B	HSD11B2	HSD11B1
JUN	RORC	ATP1A1	PTPA	GART	PPM1A	PRKCA	VDR	GLRA1	GLRA2
PTGS2	F2RL1	KCNA3	PSEN2	PSENEN	NCSTN	APH1A	PSEN1	APH1B	FDFT1
MLNR	DRD2	AKT1	MCL1	PDE5A	ABCG2	PLG	ABCB1	ALOX15	ALOX5
AHR	ESRRA	PPARG	OPRD1	OPRM1	GPR35	PTPRS	DAPK1	MPG	SLC22A12
NOX4	MAPT	KDM4E	AVPR2	TOP2A	MAOA	DRD4	GLO1	MYLK	MPO
PIK3R1	PYGL	SYK	MMP3	CA3	PLK1	MMP9	PIK3CG	MMP2	PKN1
CSNK2A1	NEK2	CA13	CAMK2B	NEK6	PLA2G1B	APEX1	AKR1C2	AKR1C1	AKR1C4
APP	CNR2	KIT	NAE1	GSK3B	PFKFB3	BACE1	CDK5R1	CCNB3	CDK6
CDK2	ARG1	TNKS2	TNKS	PLAU	FLT4	AURKC	AURKB	HDAC2	FLT1
ELANE	PDGFRA	PARP1	ACHE	CDK4	HSD17B2	CLK1	INSR	PIK3CA	HSD17B14
NQO2	CAPN1	YWHAG	WEE1	ESRRB	MTOR	BCL2	CCR5	VCP	CDK1
ERBB2	SIRT1	SERPINE1	TYMS	ADCY5	PDK1	GCGR	HSP90AB1	CHEK1	ESR1
HSP90AA1	HSP90B1	TERT	CHEK2	SCN5A	NOS2	F7	RARA	NCOA2	ESR2
CCNA2	CA7	MIF	PPARA	TBXAS1	ALDH2	SLC6A2	CDC7	PON1	SNCA
PLAT	F10	DUSP3	STS	IGFBP6	IGFBP4	IGFBP5	IGFBP2	IGFBP1	CA5B
GLRA3	GABRB3	ATP1A3	ATP1A2	PTPN2	GRM5	CES1	CES2	POLB	PLA2G2A

PLA2G5	PLA2G10	CHRNA7	KLK1	KLK2	RXRA	MET	CA6	CTSB	DYRK1B
BCHE	IGF1R	DYRK1A	SIRT2	F3	PTGER1	PTGER2	PTGER3	PIK3CB	CYP2C9
CYP3A4	PGF	VEGFA	EDNRA	ERN1	CCNE1	HNF4A	PRKCG	PRKCD	PRKCB
PRKCE	PRKCH	PRKACA	PRSS1	PRKCZ	IKBKB	TP53	TNF	SLC2A4	RELA
RB1	PTGES	NUF2	NFKBIA	MDM2	MAPK1	IL6	IL4	IL10	IFNG
ICAM1	HMOX1	GSTP1	CDKN1A	CD40LG	CCND1	CCNB1	CASP9	CASP7	CASP3
BIRC5	ADCY2	FLT3	TTR	CD38	AKR1B10	CFTR	AMY1A	GRK6	PIM1
PTK2	CXCR1	ALK	AXL	NUAK1	AKR1A1	CCR4	GPR84	PDE10A	FNTA
PDE4D	XPO1	PPP2CB	SEC14L2	DGKA	SEC14L4	SLC6A4	PKIA	ADRB2	RPS6KA5
DNM1	PTGER4	GRM2	PDPK1	CALM1	HDAC5	HDAC7	HDAC9	CNR1	PAWR
CD36	CCR7	ABHD6	MGLL	FCER1G	SUMO1	APOH	GPR55	CHRNB2	CETP
FCER1A	CAV3	RNF207	C3	DAGLA	PLIN5	ZP3	ADRB1	NCOA1	ODC1
RET	EPHB2	ALPG	ALPL	PLAA	GRM4	GSK3A	PIM2	MARK1	KDM1A
TUBB1	CCND3	ADAM17	PDE7A	FGFR2	PTK6	PIK3CD	PRKDC	HCK	MAP2K1
EPHB4	HSD17B3	PI4KB	RAF1	BRAF	PDGFRB	CXCR2	CCNE2	CSNK1G1	RPS6KA1
ROCK1	MMP14	EP300	DBF4	TRPM8	EZR	ABAT	JAK1	PDE4A	PDE4B
PDE4C	ATM	RPS6KA2	ATR	NCOR2	MMP16	HDAC11	HDAC10	CTSD	CDC25A
BMP1	ANPEP	TGM2	SPHK2	SPHK1	LRRK2	ST6GAL1	FCER2	MAP3K12	BACE2
DUT	HTR7	HPSE	P2RX3	IRAK4	LTB4R	SEC14L3	NR1I2	PDE3A	CXCR4
RPS6KB1	GLI2	HDAC1	MAP4K4	ALOX5AP	HPGDS	PGD	HDAC8	DRD5	SIGMAR1
HDAC6	VCAM1	SLC2A1	MT2A	JAK2	GABBR1	F11R	BAX	KCNMA1	CTSL
DHODH	HSD17B6	CYP11A1	WNT4	STAR	HSD17B8	DHRS9	TNNC1	NFE2L2	EGLN1
HTR3A	COMT	CSNK1D	CMA1	PIM3	SREBF2	BMP4	TRPV1	STK17B	AGPAT2
HDAC3	DPP4	ROCK2	IDH1	S1PR3	S1PR1	SCD	BRD4	MAPK8	MAPK9
NPY5R	HDAC4	FYN	OGA	CPT1A	CFD	MCHR1	PRF1	HPGD	ADAMTS5
GRIN1	TGFBR1	KDM5A	CYP1A1	CYP1A2	NTRK2	TACR2	MAPK3	TIMP1	TEP1
STAT5	SAFB	RASGRF2	PTGFR	PBK	NOS3	NCOR1	MSLN	MDC1	MAPK12

MAP2K5	LDLR	KLK3	KCNJ11	INS	IL1B	HMGCR	HIF1A	GLB1	GFAP
FN1	CXCL8	CRYAB	CDC37	CDC25C	CDC20	CCNB1IP1	CCL2	BUB1	APOA1
AHSA1	CRHR1	BAD	SLC6A3	CHRM1	ERCC5	FEN1	TRPM2	SLC5A5	RUNX1T1
PSME3	PSMD3	MS4A2	IL13	IKBKG	FXYD2	EIF6	EEF1E1	CYCS	CFLAR
CCND2	APC	ALPI	ALG5	ACACA	AAGAB	QDPR	UPP1	MTNR1A	MTNR1B
RPS6KA3	DCTPP1	CLK3	DYRK2	MMP15	MMP26	SF3B3	ADAMTS4	GUSB	MPI
ACVR1	LIPC	SAE1	STAT6	CASP6	ME1	NOX1	MPEG1	DAO	XIAP
RPS6KB2	NEK1	PLK4	CYP11B2	CISD1	SLC9A1	PPARD	G6PD	SLC22A6	FABP4
FABP3	FABP5	FFAR1	FABP2						

Table S3. The targets of allergic asthma

IL4	TNF	IL10	IL13	IL6	IL1B	IL5	CAT	NOS2	ICAM1
ADRB2	GSTP1	CYP2E1	IL33	IL1RL1	IL1RN	EDN1	ALOX5	MMP1	PLAU
ARG2	IL6R	ARG1	RNASE3	MMP10	BGLAP	PDE4D	HSD11B2	FOXP3	PPP2CA
CTNNA3	GSDMB	CLDN18	CYSLTR2	JAK2	KIF3A	ADCYAP1R1	CYSLTR1	LTC4S	SCGB3A2
MS4A2	PTGDR2	PTGIR	CCR3	TBXA2R	CHI3L1	IRAK3	POSTN	PTGDR	PTEN
FOS	ALOX5	CSF2	ALB	SOD2	CXCL1	FN1	NFE2L2	ABCB1	CYP1B1
GCLC	CDKN1A	TLR4	IL1A	BAX	BDNF	ACHE	GSR	PCNA	SPP1
AR	CREB1	NGF	IGF1	GPX1	DUSP1	IL17A	LEP	DDIT3	SQSTM1
MAPK14	TH	CLU	FGF2	TLR2	PRDX2	G6PD	LIF	CEBPB	ABCG2
ABCA1	RB1	CD86	CD44	PKM	PON1	CAV1	POMC	CCNA2	CASP8
TIMP1	MAPK8	CFTR	ABCC4	NR1I2	FOXO1	GSK3B	IGFBP3	CDKN1B	CD40
MKI67	CYP2C9	IKBKB	IL1R2	CD36	ATF3	PRDX6	H2AX	MCL1	LOX
JAK1	FKBP5	TGFB2	G=JA1	TLR3	AQP3	CYCS	HSPA1B	XDH	NR4A1
HGF	SLPI	TNFRSF1B	STAT6	ITGB2	CCR2	NLRP3	NOTCH1	C3	TIMP2
CXCL9	APOE	LCN2	HSPA8	IL3	SULT1A1	TSC22D3	FASLG	MX1	RGS4
KEAP1	CLCA1	CDKN3	CRH	CXCL12	SLC22A2	HSPD1	RGS2	GGT1	NOX4
PLAUR	CXCL3	NGFR	IRF1	CALR	MAP2K1	CDKN2B	A2M	LTF	MIR125A
COMT	EDNRB	SERPINB2	ETS2	MUC1	SDC1	EPHX1	PTX3	STAT5	ENO1
TAT	MT2A	HRAS	MIR21	PDCD4	TNFRSF9	CCL19	F2	PRDX1	COL1A2
KRT18	IL2RA	TOP2A	AOX1	SLC2A1	DNA=JB1	ADIPOQ	WNT5A	FABP5	CSF2RB
RPS6	CSF1	GFAP	PLK1	CD83	BMP2	CSF3	IL24	TAC1	TGFBI
FST	SLC18A2	HBEGF	TF	PPBP	EGF	FGFR1	PFKFB3	ARNT	DRD2
BECN1	CCND3	SFTPD	ELF3	GCG	IFIH1	TNFRSF25	TGM2	ITGA2	ITGAV
TPT1	STAT5B	LZTFL1	SMAD2	ADORA2B	FCGR2B	CREBBP	TPI1	UBC	ZEB1
MME	CIITA	BHLHE40	CTNNAL1	GAS1	PGF	CDKN1C	HSPA4	PLPP3	FPR1
CALCA	EREG	HDAC1	MX2	LEPR	ERMP1	PDGFB	RACGAP1	CCL8	CLEC7A

MSRA	PLD1	DNM1L	ITPR1	CXCL16	HSP90AB1	CD69	LTA4H	PTPRD	TEK
FEN1	CTSK	MGP	SLC22A5	PF4	SLC7A2	ITGB4	HMGCR	NPPA	CDC42
MIR222	CP	PSMD8	RAC1	PTCH1	PTPRK	RAC2	CD4	NFKBIB	TET1
ADCY9	ABL1	AGTR1	CCR5	LIFR	CCR6	IL15	PTCH1	IL11	ANXA2
SERPINA1	MYH10	IL2RB	TUBB6	SCGB1A1	GLB1	HPX	MR1	RARB	FOXA2
GNAS	SST	ACTG1	MRC1	ATP2A2	PTPRC	DAG1	ITGAX	SUCLG2	TGM1
PSMB9	CLDN7	SOX9	GZMB	APOH	DES	ETS1	MIR10A	MIR15B	LGALS1
LTB	XRCC1	PSMD12	SHC1	NOS1	ENO2	MARCKS	SIRT1	ANXA4	CEBPA
ALPP	CREB5	KL	VASP	APC	C4B	CTNNA1	MECP2	CD38	PTGDS
FBLN1	STAT5A	MIR93	SP1	CD68	ANXA6	DDB2	CD274	ESM1	LPP
IFNB1	PML	AMBP	MDM2	ADORA2A	ERBB3	CTF1	BDKRB2	NR3C2	CEACAM6
PROS1	DMBT1	ITGB8	SOS1	ADORA3	SOX2	CYP2D6	SFTPC	ADAMTS1	ADORA1
AQP4	LCK	PHB1	IDH2	PBX2	NEAT1	CHD7	IL13RA1	PSMB8	ADCYAP1
GAST	ALOX15	FGF7	BRAF	EEA1	NFIA	MICA	CLDN4	AKR1B1	CALD1
SLC26A4	ERN1	ITGA4	SUV39H1	NLRC5	CHIA	F2RL1	MGST2	HDAC5	KRT14
CTSL	LYN	PTHLH	KCNN4	SLC1A5	LPO	SCGB3A1	GRB2	PSMB1	PTGER3
CYP4F3	IL9	TNFSF13B	EPCAM	TALDO1	ADCY6	AFF4	SERPINB8	PLA2G2A	WWTR1
MIR126	GRN	SCD	FGF10	KNG1	MAPK10	PIK3CD	HSPH1	CST3	RACK1
CD151	CHAT	DDX58	RETN	SFRP2	WT1	CD27	PDE4C	ARRB2	EIF2AK2
DIABLO	KCNQ1	PSMB4	HCK	CISH	DSC1	MIR30A	CFL1	ACE	CXCR3
RPS27A	RXRB	TFDP2	TNFSF4	MAP3K1	PRDM1	HP	IL17RB	KCNE4	NFATC3
FCGR3A	GLA	U2AF1	VAPA	VTN	PSMB2	TRPM7	CLCN5	CTCF	HAVCR1
ANK2	CAMP	DDR1	NTF3	AXL	HAVCR2	MIR149	VDAC1	ASIC1	TREM1
COL18A1	NAT2	B3GAT1	CD58	COX5A	DUOX2	LTB4R	ACVR1B	FCER1A	CD163
GP1BB	PMS2	TNFRSF10D	WLS	CYFIP2	SOAT1	SLC8A1	KCNQ1OT1	EIF2B5	GALNS
ITPR2	BPIFA1	HDAC6	IDO1	NPHP3	SGCE	CCL26	TFAP2A	CES1	ITGA1
CYBA	PLA2G6	SPTBN2	CD59	ADAM33	MIR142	MIR183	PSMA3	BCL2A1	MIR143

MIRLET7C	DEFB1	SERPINB8	SLC7A1	ZFP36L1	IL25	MYLK	ABCB9	NPHP4	NPTN
MIR146A	HDAC2	GNA13	MYH11	SRSF3	WNT3A	MUC21	PROC	FADS2	S100A7
HTR3A	HYKK	CTTN	HDC	IL22	PTPRM	ARHGEF2	CHEK2	FAM13A	RPS6KA5
SIGIRR	SIK3	SEC14L3	RAMP1	SAMHD1	TSHR	ACVRL1	ACE2	NKX2-1	TAF7
RAD51B	SRP72	PNMT	ORAI3	SLC30A4	ACSL3	GNRH1	PRKCZ	ANGPT2	PDXK
NOD1	MAG	RIPK1	ACTC1	PDE5A	TLR10	ADCY1	H2BC21	ITGAL	PLCG1
POU2F1	YY1	MMP28	CHRM2	F10	LGALS8	FCGR2A	KCNA2	PSMA5	MUC16
IL5RA	MUC4	IL13RA2	CR1	ADH1B	HHIP	NEIL1	TRAF3IP2	INHA	OSM
EP300	ERBB4	HSPG2	S1PR3	TSC2	RET	PLA2G5	ADAM10	SEC61A1	TTN
CDSN	ALOX12B	BMPR1A	EDN2	FCGRT	FNIP1	IFT80	TRAIP	USP10	CLIC1
COL4A3	LACTB	NACA	CYYR1	DERL2	LST1	ADAM8	PEPD	TPD52	APOBEC3B
CCL21	H2AC20	MIR574	CRHR1	MFAP4	CARD10	GDI2	SPINT2	TRPC3	ADGRB2
MST1	RIPK2	SCT	SFRP4	TOLLIP	ODAD3	PTGFR	SCNN1B	DERL1	RANBP2
SEMA3A	MIR145	PRG2	PRTN3	NRAS	ADAM12	DSG1	PAF1	PSMA2	ALOX12
WDR46	ARL6	KCNMB1	KLK5	SUOX	ILF2	ORAI1	RPS6KA3	CD48	EVPL
NSD1	WIPF2	KAT5	KCNMA1	ARSA	PADI4	PCDH1	SETD7	CYP3A7	CDON
H4-16	MIR206	RTEL1	PPP1R12A	PAPPA	SOX18	GRP	TNFSF13	ITIH3	CAPSL
TYR	NTRK1	NFATC2	IFNA1	INSIG2	DYNLT1	ELOVL4	ENG	RFX1	ZDHHC1
IQGAP1	MTHFR	H2AC18	ADAMTS12	MRPS31	PEDS1	SUFU	ABCA3	GC	CD276
EDN3	CD79A	KMT2D	ITIH2	LSR	RGS5	CHRM1	SPATA13	DISP1	PDCD1
IL19	MIR152	EHBP1L1	HGS	KRT20	TNFRSF8	YWHAB	FGF8	BCL11B	ADRA1A
BBS1	IL17D	PI3	CEACAM3	DOCK8	TAPBP	TMPRSS11D	ITGB3	PTPN1	MIR34A
MIR140	RNF5	SEC24C	TTC38	ZFYVE9	EHF	SMURF2	SOCS5	PAFAH1B1	IFT140
RFX5	CARD16	CDH26	IL31RA	TMSB10	NPPC	CHML	MIR144	SPINK5	SPRR1B
GHITM	G=JC1	IFNAR1	NODAL	UNC119	DEFB4A	P2RY6	BRD4	MAP3K11	SERPINB3
CIAO3	MAP3K13	ERAP1	HNRNPM	HLA-DPA1	ITCH	MYRF	SKP1	MIR139	ZNF331
P2RY12	ELF5	ZIC2	GIPR	RAB5B	STXBP1	TSC1	PTPA	SIPA1	BUD23

MIR638	PLA2G1B	PPIG	RRP1B	ORMDL1	STK16	SETD2	DAP3	F2RL2	CD1A
PTTG1IP	CRKL	IRGM	MKNK1	CHIT1	P2RY4	TRPV4	ZBTB8A	IL17C	MIR15A
MIR424	RIC1	AIMP1	ADPRH	SNW1	HTR4	MIR214	RNASEH2C	MIR148B	CLCN4
MIR335	TICAM1	DPP10	SMC3	MIR124-3	KLK1	CIB1	ELOA	KIF7	OPN3
CASR	PRKG1	AP2S1	P2RY1	RPGR	TEC	TUG1	DNASE1L3	GABRA3	CMA1
RASGRP4	HLA-E	PRSS21	LNX1	SGCB	AKR1A1	TRPM8	IL37	ZCCHC10	NR2C1
CD300C	PRG3	TERC	TGM3	FAF2	FUT2	LMX1B	MADCAM1	SPEF2	HRNR
CTRL	MAML3	SMARCC2	UPK2	IL17RC	SLC24A2	AVPR1B	DCDC2	UFD1	PLD2
RTP3	SPOP	ZFPL1	SLURP1	TPSG1	SNX32	ZNF77	WAC	C3AR1	CCL28
BRS3	CCL23	FENDRR	TRPC4	GTF2H5	SCN9A	MMP25	DNAH9	UTS2	TACR1
COG6	GNB1L	SPTLC1	MRPL41	NEK9	MYLK3	SIX3	GABRA2	MRGPRX2	TMEM165
GP9	CCR10	MIR499A	UPK3A	ZNF365	FGFBP2	UBAC2	MIR218-1	NPS	RTRAF
TDGF1	LALBA	VPS51	IFNA2	SULT1A3	CRISP3	AAA1	GPR12	TINCR	SPOUT1
ATP5MG	MIRLET7A1	MIR216A	RABL2B	FCGR2C	CEACAM8	CDKN2B-AS1	MS4A3	DEFA1	MTMR9LP
MIR320C1	HCG23	IFNL1	MIR19B2	MAP3K14-AS1	MIR620	DEFB103A	MIR3936HG	TRA	HCG22
TGFB1	CCL2	VEGFA	CCL11	CD14	MMP9	BCL2	HMOX1	GSTM1	CCL5
AREG	ALDH2	HNMT	PARP1	TIMP3	STAT4	PDE4B	AGER	TBX21	TSLP
HTR2A	PLA2G7	NPY	MYB	HLA-DQB1	HLA-DRB1	ADCY2	CTLA4	RAD50	TRPA1
DNAH5	HLA-DPB1	NPSR1	PYHIN1	CDHR3	PRKD1	PTGS2	MUC7	PLA2G4A	PRKCA
IFNG	CXCL8	MAPK1	MAPK3	IL2	MPO	CASP3	RELA	CYP1A1	NFKB1
NFKBIA	PPARG	FAS	IL12B	AHR	NR3C1	CXCL2	VCAM1	TP53	CXCL10
CASP1	NOS3	EGFR	AKT1	CCND1	CTNNB1	MUC5AC	CYP3A4	ACTA2	TNFRSF1A
TNFSF10	PPARA	CCN2	ODC1	SOCS3	CYP1A2	ABCC2	NFIL3	ITGAM	GSTT1
ADM	GSTM3	COL1A1	MGST1	MMP2	S100A9	BCHE	STAT1	ABCC1	HSPB1
PGR	SMAD3	TNFAIP3	HSPA1A	CASP7	CCL4	MBP	HIF1A	GPX3	TGFA
MAPK9	THBS1	ITGB1	HSP90AA1	CTSD	IRF7	CASP9	IRS1	SLC22A1	CXCR4
IL12A	BCL6	NFKB2	CYP3A5	GPT	HSPA5	S100A8	CDKN2A	CEBPD	CRP

MET	CCL7	GSTA1	ATM	SELE	FOSL1	PLAT	CDK2	MYD88	CASP6
IL1R1	NTRK2	TFRC	IL6ST	CXCL5	PRKCD	MMP13	ISG15	TGFBR2	AKR1C3
RHOA	IL7R	FGF1	IGF1R	MMP3	TTR	CCNB1	VIM	TGIF1	IL4R
LMNA	NFKBIZ	SLC22A3	C5	EPX	ALDOA	TGFBR1	UGT1A1	LEF1	INS
GATA3	GSTA2	TNFRSF12A	SELL	INHBA	GSN	TNFAIP6	IL23A	BIRC5	SRC
DUSP10	IGF2	MMP8	IL15RA	MAF	CDK1	KLF2	STAT6	TRAF6	PRL
BIK	IFI27	RPS6KB1	ADRB1	CTSB	SCNN1A	BAK1	XBP1	BAG3	PECAM1
FOXO3	PRNP	PRDX5	RUNX2	SLC22A4	KIT	PDGFRB	REN	SYK	NT5E
ATG5	FCER1G	C1QBP	THBD	NFATC1	BRCA2	TNFRSF10B	CCND2	MALAT1	AKT2
SFRP1	CXCR2	DUSP6	CCL24	F2R	TGFB3	CD80	SMAD1	TRAF3	HMGB1
CX3CR1	FOXA1	CSF3R	SLC9A3R1	ACTA1	HSD11B1	ANPEP	MMP14	EPO	GATA4
MAPKAPK2	EPHX2	TAP2	TNFSF14	S100B	TXN	TXNIP	F5	ERBB2	CEACAM1
MMP12	HMOX2	CLDN1	EZR	CCR7	ADA	SPHK1	VWF	LTBR	SOD3
ICAM2	MIR155	MTOR	UCP2	TAGLN	OAS1	HMGA1	CTSC	SNAI1	ADRB3
AKR1C1	MBD2	MAP2K2	PDGFA	CD74	CDH13	ITGA3	PRLR	TYK2	HPGD
PROCR	PPARD	SPARC	VDR	IFNGR1	TNFSF12	ACLY	TLR1	KRT10	GUSB
IDE	STIP1	XIAP	IVL	IL16	MAP2K3	GAS6	PRKCE	CD9	CFH
NRG1	HPSE	EPHA2	PTPN11	CHEK1	RAF1	CXCR6	SERPINB1	BMI1	CNN1
LBR	HSP90B1	MB	LAMB3	SERPINB5	MAOA	TNFRSF10A	SPTAN1	VCL	HYAL1
LGALS3	TKT	NR0B2	BMP6	MYH7	ALOX5AP	ACP5	RORA	RORC	CCL1
ALDH1B1	HMGA2	TLR5	CALM1	PLCB1	FLT1	BMPR2	GLDC	ATG7	SMAD7
DMD	PPL	SERPINE2	KCN=J11	PSMD14	PHF11	PRKAA2	CFLAR	CCL17	SFTPB
BMP4	CD93	ENTPD1	MEG3	P2RX7	IL32	ATF6	AGR2	CD40LG	F8
PSMD7	FPR2	KDM6A	NOTCH4	GHRL	COL2A1	DCN	CYP2=J2	FANCC	TPO
ADAM17	GLI2	KRT13	ECI2	SMAD6	MT1X	LMNB1	NPPB	ICOSLG	MS4A1
WFDC2	CCR1	ECE1	TUBA8	MIR98	NPR3	PDE4A	PTGER4	PLEK	CCN4
ALDH9A1	LTA	PFN1	ROBO1	TPM2	ERCC1	FHL2	ELK1	PDGFRA	ARSB

DPEP1	NPM1	ZFP36	EEF1B2	PTPN6	ROCK1	SHH	APEX1	LRG1	PAM
XPO1	SYP	ACTN2	MIR132	PRKN	SPON2	FYN	HSPE1	PDCD5	KDR
GNA12	CANX	LBP	PTK2B	CADM1	ELN	ACKR1	WDR19	KLF6	AOC1
NCOA2	LTBP1	MIRLET7D	MIR200B	PTGES	TSPO	KDM4B	PSMD1	RAPGEF3	IL1RAP
TNFRSF10C	CBL	HBB	HLA-A	KPNB1	TBL1XR1	P4HA2	KRIT1	LAMA3	MIR19A
ITPR3	PARK7	PSMA4	TGFBR3	PCDH12	F12	PPIA	EDNRA	SEPTIN9	CALM2
0	KITLG	ERCC6	PXN	HLA-C	PSMA7	PSMA1	YWHAZ	RREB1	STING1
PDE3B	SATB1	PIM1	SLC9A3R2	VCP	HAS1	HDAC8	ENPP3	FGR	YBX1
ICOS	TCF7	GLRX	CUL1	CSTA	PDGFC	TFAM	SMARCA4	GGT5	IL17F
SERPINA6	YAP1	AQP5	SPATS2L	CNR1	ADH5	PDE3A	PSMC2	KCN=J5	MUC5B
IL10RA	BMPR1B	EHD1	ITGB7	ANGPT1	ESRRA	EIF4EBP1	CTSS	ELAVL1	PIK3CG
LILRB3	NR1H2	FAM117A	LILRB4	ELAC2	TRPC1	CD28	BDKRB1	IRF3	VDAC2
PLCG2	SFTPA1	GREM1	SMARCA2	MIR34B	TMPO	SPTLC2	BSG	CAST	PSMB5
TMEM79	TPMT	CXCL6	PIN1	KLF15	GAL	NOX1	LAMC2	CETN2	IL18R1
VCAN	MAP1LC3A	RBM14	ELL	CD8A	CHRM3	HRH3	MIR148A	FCGR1A	MIR27A
LPCAT2	ERCC2	LGALS3BP	PAWR	TP63	LAMA1	EIF4E	CCDC28B	ACVR1	CDK12
IDS	RPLP2	TAX1BP1	IVNS1ABP	CYP2A6	NTS	ARPC2	SMCHD1	TINAGL1	GRK5
OPRM1	LTBP4	CTSG	MIR185	P2RY2	IRF4	LGALS7	SART1	SCNN1G	ATP8A1
MIR22	DUOX1	GLP1R	THSD4	COL26A1	CTSW	KRT16	STX1A	GABBR1	ACTN3
CHRNA5	CXCR1	HPGDS	MMP19	XRCC3	ESYT1	RHOD	BMP7	ANO1	MUC2
SERPINB7	TBX5	KLF10	PRKDC	FUT3	SLC26A6	TTF2	RUNX3	CXCR5	FAIM2
PSMC3	VIP	MYO18A	RAB11A	NOTCH3	CHGA	MIR29A	TBCK	MCAM	RTN4
CEACAM5	EDC4	MUC6	PON2	FOX=J1	IL17RA	ATP12A	AVP	KAT7	ADRA2A
DDX39A	HNRNPA2B1	SERPINA7	SMAD4	TLR7	TXN2	HLA-DRA	LAG3	KLK7	RNASE2
CD70	PLCB3	SCN1A	SELPLG	ANK3	USP7	CYP24A1	PLAG1	CSF2RA	LIG4
AMFR	CD247	DDR2	MAP3K7	PRSS8	ELP1	GOPC	PIK3CA	ALK	CLDN2
TFF2	GNAI1	FURIN	SLC12A4	TLR9	CD5	MIR192	MIR223	MIR31	SLC35C1

ANK1	ITIH1	STIM1	PRMT7	TNFRSF4	TULP4	GAB1	PTGER1	SLC44A1	GHRH
CHRNA3	ATP2A3	PDE7A	S100A12	CD34	EPHB2	HDAC9	SIRT2	TRIM25	ZFR
SERPINB10	CNTF	FLG	STUB1	MYH9	TRAPPC3	CALM3	AANAT	CLTCL1	KIAA1109
C5AR1	CCKBR	CXADR	DUS2	SLC25A46	MIR30B	FTL	SPHK2	TLR6	ICAM3
PLCB4	ABCB7	EHMT1	ERLIN1	SRF	ADCY10	MYDGF	TACR2	UBE2Z	RBP4
DDX1	MED1	PLA2G3	PLA2R1	TMEM67	IRF5	MCC	SLC26A2	ORMDL2	PVT1
NUDT6	FSIP1	NSUN7	SYVN1	VAMP2	MC1R	MIR221	HERC5	LGALS9	SLC27A4
UNC45A	CLCN2	IL12RB1	THSD7A	CHRNA7	AGK	MKS1	TXK	LSM4	ALMS1
IL27	TNFAIP8L2	TPSAB1	UNC13D	CD160	ZNF614	ADH1C	MAVS	SPI1	TNFRSF13B
PCDH8	OPRK1	AKAP13	CLCN3	DDX41	IPO13	NEU1	PMEL	SIGLEC5	SSTR5
FSTL3	GLCCI1	HAGH	PSMD3	WNT7B	DOK1	HLA-B	ANAPC1	IRF2	NIPBL
AHSA1	HRH2	SLC12A2	CCL25	STOML2	RAB5A	BPI	CD1D	EMX2	SPRY2
CLEC12A	ODAD2	SLAMF8	SLC26A9	ATPAF1	MID1	MYL9	IL20	UBE3C	SCG5
GP1BA	PLA2G10	SCN2A	NCBP1	TSPAN8	CXCL17	ELANE	MIR29C	SNRNP40	ATR
CMKLR2	HRH4	CLC	PGM3	MGAM	MSLN	COX4I2	CCL15	CRLF2	CCR8
MMRN1	NTF4	TPSB2	MGA	GATA1	MIR24-1	KCNN1	ADAMTSL1	MRPL11	PNOC
CCL27	F2RL3	TRPC6	IL1RAPL2	SERPINB4	TAC3	MIR9-1	MSH5	SEZ6L	TACR3
TM9SF2	CYP4F22	SNTB2	ZMPSTE24	LORICRIN	FGF14	PPP2R5D	COL16A1	RIOX2	ARPC3
MIA3	SDHD	MUC3A	RETNLB	FLG2	DENND1B	NCKAP1L	OTULINL	SPTSSA	TFF3
H3C14	SFTPA2	OIP5-AS1	PDCD1LG2	RMRP	SCN4A	SPDEF	CALB2	TRPV1	LYNX1
MIR29B1	CLEC16A	SETDB1	KLHL22	LTB4R2	AICDA	CCHCR1	BTNL2	EFEMP2	MIR204
PSMD4	TCF20	GLO1	PEX5	TUBB1	ADGRG6	UCN	HIRA	ZNF432	PPP2R1B
TNXA	CPQ	CCL13	ART1	AGMAT	IGHE	SPECC1L	CARD11	GUCA2A	MIR133A1
ZNF699	CTSE	PEF1	HYAL3	OXA1L	STAU1	CNTROB	LILRA2	MIR203A	SNX27
HYAL2	FCHSD1	ARSL	HLA-DQA2	PSMA8	POU2AF1	CARMIL2	FOXH1	CFAP95	GUCA2B
KIR2DL4	CSN1S1	CEP89	LELP1	TSBP1	RANBP10	GRHL2	IDO2	KLK11	MIR92A1
MIR124-2	NMUR1	DEFB4B	MIR548AN	CLEC6A	SNHG5	TAS2R1	SIGLEC8	MIR625	DEFB103B

ARSH	FAM81B	MIR125B2	FCRL6	SPATA32	MIR125B1	CA10	MIR217	HLA-DOA	RNU4ATAC
MIR19B1	ZNF841	MIR124-1	SOD1	KRT19	HLA-G	PTGS1	CCL3	MYC	EGR1
NPSR1-AS1	MIR629	MIR3619	NQO1	TNC	ORMDL3	PTGER2	ESR1	GCLM	CCL20
PPARGC1A	STAT3	UGT1A6	ESR2	SELP	CYBB	ANXA1	AGT	GAPDH	BCL2L1
IL18	ABCC3	CDH1	SERPINE1	MIF	PIGR	RARA	LY96	APOA1	F3
DLC1	HDAC4	CX3CL1	DKK1	IFRD1	MARCO	DPP4	CD63	SRSF2	IGHE
TAP1	PTAFR	GSTO1	ITGA5	IL7	CXCL11	E2F1	PRMT1	CHUK	TERT
GSS	GATA6	CIRBP	THRA	SEMA7A	LY86	MGST3	PSMC4	CYP2C19	PDIA3
CASP4	HRH1	CCL22	ENPP2	CLDN5	SERPINA3	CD55	FGFR2	RANBP1	NAT1
NRF1	SCG2	NTRK3	CASP10	PSMC6	ALG9	MMP7	CREG1	TMPRSS2	PSMC5
PTK2	BRCA2	PSMD11	ANXA5	RYR1	FCER2	DLL1	SEMA3E	IFNGR2	SLC9A1
NBR1	SMARCE1	PSMB3	DNA=JC5	NSUN2	MIR20A	PTPN3	PKDCC	CIZ1	GSTO2
ST6GAL1	TNFRSF14	FAM167A	SOCS1	SLC6A14	MAP3K5	ARF4	CLIC6	MKKS	MDC1
CDX2	SMOC2	IKZF4	CTNND2	PSIP1	TAGLN2	MDN1	FOXC2	EML4	PDPK1
MIR200C	CENPT	IL12RB2	NOD2	TPR	CGN	IRAK1	LRBA	PSMA6	TBX1
CHKA	CCR4	ITK	MYH14	SCYL1	CSN3	CALN1	ITGA2B	TRAPPC10	TOR1A
CPA3	ITGAE	ZNF217	BRD2	ACP1	HBG2	COPA	MBL2	CCK	EDA
IPO8	SOCS6	IL9R	CCR9	PYY	EPRS1	ARVCF	MEN1	CD46	PABPN1
CYP27B1	GSDMA	LCN1	MIR17	SMPD1	GMPPA	CCL18	ST8SIA2	BTK	FOXA3
IL31	MSMB	CACNG7	DNASE1	FCGR3B	DCTN4	MIR320C2	TSSK3	RLN1	LINC01924
FCAR	ROM1	LAYN	HAX1	TSGA10IP					

Table S4. Core targets of JGT in allergic asthma treatment

AKT1	TNF	TP53	IL6	VEGFA	INS	EGFR	SRC	IL1B	STAT6
MAPK3	CASP3	HSP90AA1	HIF1A	ESR1	PTGS2	FN1	ERBB2	CCND1	MMP9
MTOR	CXCL8	PPARG	IL10	MAPK1	BCL2L1	CCL2	NFKBIA	SIRT1	NOS3
EP300	MDM2	RELA	PIK3CA	MAPK14	ICAM1	JAK1	IL4	IL2	CXCR4
PPARA	HPGDS	KDR	HMOX1	PTK2	NR3C1	HSP90AB1	MPO	IL13	PRKCA
FYN	F2	PRKCD	CYP3A4						
